# Predicting target profiles with confidence as a service using docking scores

**DOI:** 10.1186/s13321-020-00464-1

**Published:** 2020-10-15

**Authors:** Laeeq Ahmed, Hiba Alogheli, Staffan Arvidsson McShane, Jonathan Alvarsson, Arvid Berg, Anders Larsson, Wesley Schaal, Erwin Laure, Ola Spjuth

**Affiliations:** 1 Department of Electrical Engineering and Computational Science, Royal Institute of Technology (KTH), Lindstedtsvägen 5, 10044 Stockholm, Sweden; 2 Department of Pharmaceutical Biosciences, Uppsala University, Box 591, 75124 Uppsala, Sweden; 3 National Bioinformatics Infrastructure Sweden (NBIS), Department of Cell and Molecular Biology, Uppsala University, Box 596, 75124 Uppsala, Sweden

**Keywords:** Predicted target profiles, Virtual screening, Drug discovery, Conformal prediction, AutoDock Vina, Apache Spark

## Abstract

**Background:**

Identifying and assessing ligand-target binding is a core component in early drug discovery as one or more unwanted interactions may be associated with safety issues.

**Contributions:**

We present an open-source, extendable web service for predicting target profiles with confidence using machine learning for a panel of 7 targets, where models are trained on molecular docking scores from a large virtual library. The method uses conformal prediction to produce valid measures of prediction efficiency for a particular confidence level. The service also offers the possibility to dock chemical structures to the panel of targets with QuickVina on individual compound basis.

**Results:**

The docking procedure and resulting models were validated by docking well-known inhibitors for each of the 7 targets using QuickVina. The model predictions showed comparable performance to molecular docking scores against an external validation set. The implementation as publicly available microservices on Kubernetes ensures resilience, scalability, and extensibility.
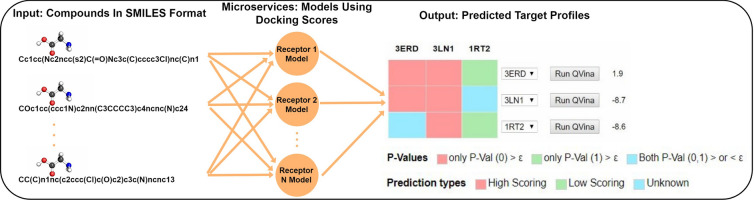

## Background

Determining ligand-target binding is a vital part of the drug discovery process [1]. A ligand can bind to multiple target proteins [2] and may cause off-target effects [3, 4]. Knowing the off-target effects of drugs can be beneficial especially in the initial stages of drug discovery. To determine drug-target interactions, pharmaceutical companies and academic institutions involved in drug discovery apply different techniques to detect drug-target interactions, including in-vitro pharmacological profiling [5]. However, another interesting method is to build in-silico target profiles for ligands [6][7], which helps in understanding off-target effects as well as providing a novel opportunity to predict affinity of Novel Chemical Entities (NCEs) against a battery of targets.

A common method to construct target profiles is to predict them using QSAR models based on interaction values available for known active ligands in large interaction databases like ChEMBL [8] and ExCAPE-DB [9]. Yu et al. [10] presented a systematic approach for predicting drug-target interactions from heterogeneous biological data employing Random Forest and SVM. TargetNet [11] is a web service for making prediction based drug-target interaction profiles using Naïve bayes based multi-target SAR models. In TargetNet, the molecules can be predicted against 623 SAR models. Bender et al. [12] employs Bayesian based technique to prepare seventy QSAR models that were used to create target profiles to predict adverse off-target effects of drugs. TargetHunter [13] is another web-based tool for predicting target profiles employing chemical similarity where the models were trained on ChEMBL data and successful predictions were made on examples taken from PubChem bioassays. The polypharmacology browser [14] is another web-based tool for multiple fingerprint target prediction primarily based on ChEMBL bio-activity data.

A key disadvantage with QSAR based modelling studies is their dependence on experimental data from the large interaction databases. Normally, the data has a strong bias towards active compounds i.e. on-target or intended effects [15]. Based on this, it is counter-intuitive to use ligand’s on-target binding data to build target profiles for understanding off-target effects. So when studying adverse target reactions it becomes beneficial to find another way than to just look at data from the databases. Furthermore, in some of the earlier research efforts, openness of the source-code and extensibility of the web services is not completely clear.

Another approach is to build models from molecular docking scores using a docking software and perform ligand predictions using the models. In [15], LaBute et al. presented an approach to predict adverse drug reactions using scores produced by large-scale docking on High-Performance Computing machines. AutoDock Vina was used to dock 906 ligands out of which, 560 conformers were selected to train L1-regularized logistic regression models to predict 85 off-target effects. Similarly, Wallach et al. [16] presents a method for logistic regression based model training using docking scores from eHiTS [17] docking software for predicting side effects of drugs. Building predicted target profiles based on docking scores is less common because the docking scores are not considered to represent the real drug-target affinity, but large training datasets allows to make better decisions and can cover this weakness.

One important limitation is lack of information about confidence on the predictions in both of the above mentioned approaches, i.e., ligand-target interaction based QSAR models and docking scores based models. Confidence on predictions are of critical importance because off-target drug reactions can directly effect human health.

**Fig. 1:**
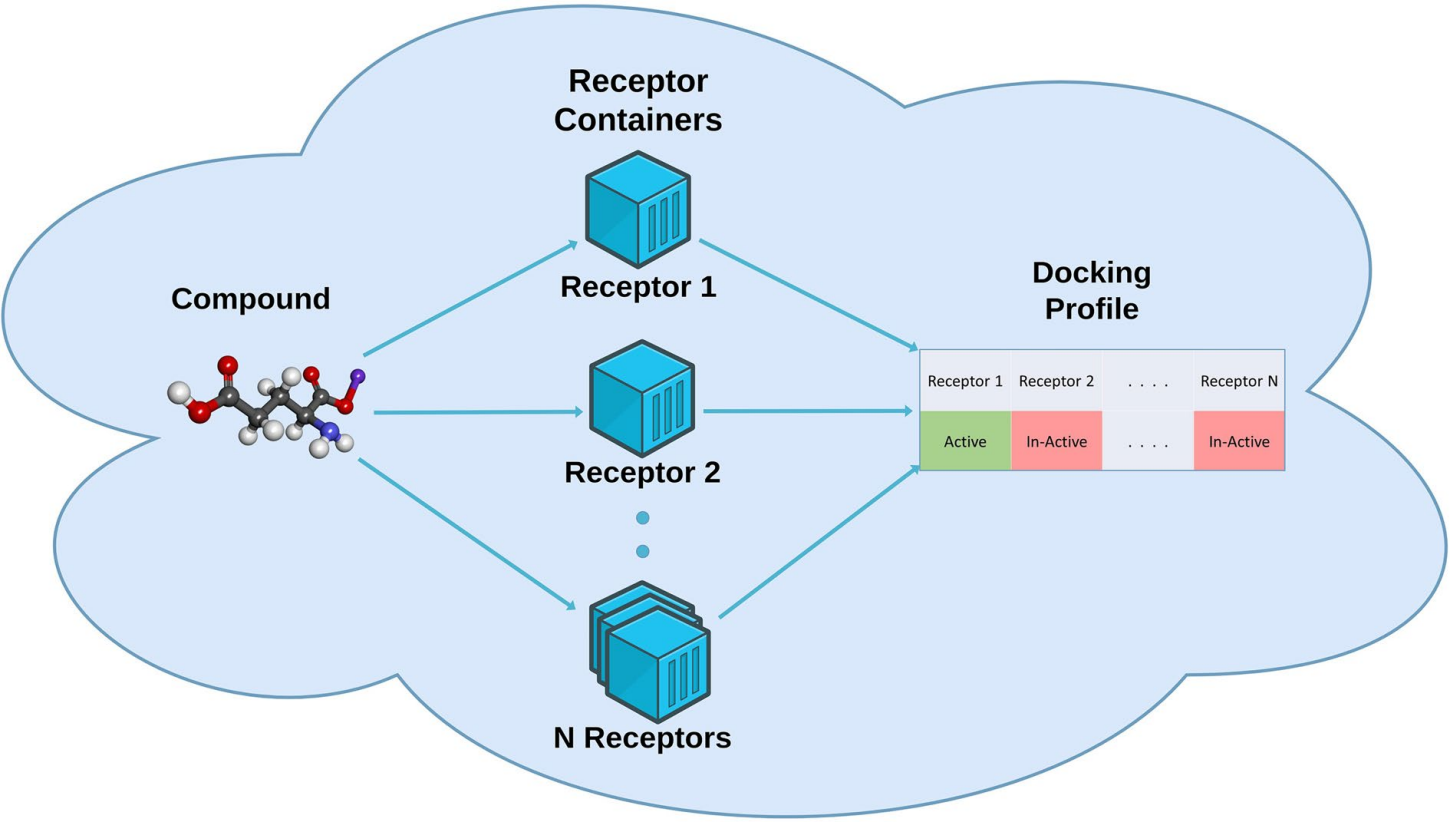
Vision of the work. The figure shows the vision of the work i.e. all targets would have a Docker container and these Docker containers would be fired up simultaneously in a Cloud environment. A compound of interest would be tested against all the targets and a target profile of the compound would be created

In this paper we introduce an extensible methodology for predicting target profiles with confidence, where models are trained on docking scores. The methodology is implemented using a microservices architecture with each target deployed as a Docker container (see Fig. 1). For orchestration we use Kubernetes managed by Rancher [18] providing resilience and scalability. The result is an open-source extendable web service, and we demonstrate it with a panel of 7 targets where models are trained on QuickVina docking scores. We also show in this manuscript that target profiles built using docking scores has predictive properties, and that conformal prediction enables quantifying the confidence for each target in a panel.

## Methods

### Data and tools

We used the *clean drug-like molecule library*, downloaded from ZINC [19] in ready-to-dock SDF format, preprocessed according to the protocol in [19]. Two distinct datasets of $\sim$2.3M molecules and 200K molecules were randomly sampled from the *clean drug-like molecule library* as the modelling set and the validation set respectively. The modelling set was used for modelling and internal testing and the validation set was used for external testing. The molecules were described using the signature molecular descriptor [20]. A parallel signature descriptor [21] implementation with Spark was employed and consecutive signature heights of 1–3, i.e., an atom at a distance of max 3 edges, were used. An earlier study [22] identifies that signature heights of 1–3 works well with Support Vector Machine (SVM) [23] based molecular classification. A fast version of Autodock Vina [24], i.e. QuickVina 2 [25] was used as the underlying docking tool.

The 7 targets 1RT2, 1E66, 1QCF, 3ERD, 3LN1, 1BNU, 1B8O were selected from the safety-related targets in [5] based on availability of good 3D structures for docking and known inhibitors. The PDB entry for each target was selected based on high resolution, i.e., 2.5 Åor better [26]. Receptors and binding site information were downloaded from sc-pdb [27] database and receptors were prepared using OpenBabel [28]. Each receptor was docked and scored against its ligand from the receptor-ligand complex using root-mean-square-deviation (RMSD); an RMSD below 2.0 Åis considered to be a successful docking [29]. Table 1 presents the final set of receptors, their PDB codes, resolution and RMSD against corresponding ligand.

**Table 1:** Selection of receptors: the table represents the selected receptors and how they were selected

Target class	PDB entry	Resolution (Å)	RMSD (Å)
HIV RT	1RT2	2.5	0.46
Acetylcholinesterase	1E66	2.1	0.34
HCK Tyrosine kinase	1QCF	2	0.29
Estrogen receptor	3ERD	2.03	0.57
Cyclooxygenase-2	3LN1	2.4	0.27
Carbonic anhydrase 2	1BNU	2.15	1.21
Purine nucleoside phosphorylase	1B8O	1.5	0.37

All the selected receptors must have resolution of 2.5 (Å) or under and RMSD of 2.0 (Å) or under

A set of well-known inhibitors for each of the receptors was compiled for testing purposes. The inhibitors were selected by reported affinity and downloaded from CHEMBL [8] and Drugbank.ca. [30] The average number of inhibitors in each set was $\sim$50 with the minimum at 43 and maximum at 60 inhibitors. A set of 50 compounds with low affinity for one of the receptor with PDB-ID 1BNU was also downloaded from CHEMBL for testing purposes. A large number of less active compounds were found for the receptor 1BNU and therefore, it was the main target used for the cross reactivity. For a list of all the compounds used in the study and a comparison of the known active and inactive compounds for 1BNU, see Additional file 1.

### Conformal prediction

Conformal prediction is a mathematical framework proven to produce well calibrated predictions for given confidence levels, developed by Vovk et al. in [31]. Instead of producing point estimates as most traditional learning algorithms, Conformal Prediction instead produces prediction regions or prediction sets. In classification the predictor outputs confidence p-values for each class, which together with the user-defined confidence level produces the final prediction set. In the binary classification setting, classes 0 and 1 translate into four possible prediction sets {0}, {1}, {0,1} and Ø (the empty set). The prediction sets are guaranteed to contain the true label of the object with a probability equal to the user-defined confidence level. For this guarantee to hold, the only assumption is that the observed data is exchangeable [32]. Knowing that Conformal Predictors always produce valid predictions, one only has to care about the efficiency of the predictions. The efficiency of a Conformal Predictor can be defined and evaluated using various metrics, see [33] for a thorough discussion on the most commonly used. We here define efficiency as the ratio of single-label prediction sets.

In this work we are using Inductive Conformal Prediction (ICP), that works in the following way; training data is randomly partitioned into two disjoint sets called *proper training set* and *calibration set*. The proper training set is used to train the underlying learning model. The model is then used for predicting all observations in the calibration set and a *nonconformity measure*, a ‘strangeness measure’, is used for computing how *conforming* each observation is compared to the learned model. We use a Mondrian approach that treats classes individually and has been shown to have beneficial properties when working with unbalanced datasets [34]. It is important to point out that conformal prediction delivers individual prediction intervals for each object predicted, and hence each prediction incorporates a measure of its confidence, implicitly offering a solution to the fuzzy concept of ’applicability domain’ [35]. For further details on conformal prediction and its use in QSAR, we refer to previous studies [32, 36].

### Modelling

For building the machine learning (ML) models, we used our earlier work, an intelligent iterative conformal prediction based virtual screening (CPVS) [37] strategy. A modified version of CPVS was used for modelling, whereas QuickVina [25] was used for docking. CPVS is an SVM based, efficient, parallel, iterative virtual screening method. QuickVina is an opensource tool and therefore permits inclusions in web services to be used by everyone. In QuickVina, a ligand with a lower score is generally considered to have better affinity against a particular receptor, therefore, the labelling strategy in CPVS was modified accordingly, i.e., ligands with low scores were labelled as 1 (high-affinity) and ligands with high scores were labelled as 0 (low-affinity). A sample dataset was docked and sorted by docking scores and the top 10% and the bottom 10% of the molecules were used for model training. The rest of the strategy was same as given in the original CPVS method [37]. The model training was performed in an iterative fashion until the model reaches the intended efficiency of 80 or above. During modelling, an average of $\sim$0.53 million ligands were docked against each of the 7 receptors. In comparison to the mentioned studies (see Table 2), the training set for modelling in our study was much larger, i.e., on average $\sim$0.11 million ligands per receptor model. Each trained model was deployed as a Docker container with a REST API.

**Table 2:** Training data size in earlier studies

Study	Average training data per receptor
Yu et al. [10]	5415
TargetNet [11]	175
Bender et al. [12]	1432
TargetHunter [13]	216.6
Polypharmacology browser [14]	33.5
LaBute et al. [15]	906
Wallach et al. [16]	1236

### Web service

**Fig. 2:**
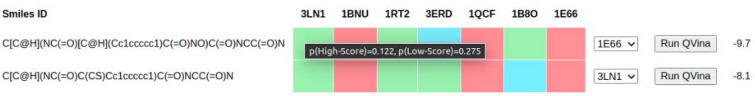
Predicted profiles and molecular docking. The figure shows the predicted target profiles for two compounds against 7 receptors. The prediction is either low-scoring, high-scoring or unknown presented in green, red and blue color respectively. The prediction models were developed based on QuickVina docking scores. Following QuickVina, in general, a low-score prediction means high-affinity and vice versa. An unknown prediction means the model has either failed to recognize a class for the compound or the compound is predicted to be part of both classes with the given confidence level. The p-values for the low-score and high-score class are also available by hovering over the prediction cells, seen here in the black placeholder. A molecule of interest can then be docked against a particular receptor using QuickVina

We developed a Web service with a front-end that offers a graphical user interface (GUI) to input one or more chemical compounds in SMILES format and options to set the confidence level for predictions. The GUI communicates with all individual target model microservices, and delivers a panel of target predictions; HIGH, LOW or UNKNOWN docking score. The predictions are based on conformal p-values, i.e. if only p-value(0) $>~\epsilon$, then the output prediction is HIGH, if only p-value(1) $>~\epsilon$, then the output prediction is LOW and if both p-value(0) and p-value(1) are greater or less than  $\epsilon$, the prediction is UNKNOWN, where $\epsilon ~= 1$ - confidence. An example of the predicted target profiles for two compounds is shown in Fig. 2. For QuickVina, a low-score prediction means high-affinity and vice versa. The actual p-values for the low-score and the high-score classifications are available by hovering over the prediction cells.

Once target profiles are produced, the user can select individual compounds and invoke the molecular docking functionality to dock them. The time for docking a compound varies between 10 to 30 seconds on our system. We also provide a functionality for users to submit new receptors in PDBQT format to the system administrator and request inclusion in the system. This requires quite some work, and will be done as time permits.

#### Implementation and deployment

The REST API for the web service was implemented using microservices and the Play 2.0 [38] web application framework using Scala language and deployed using Rancher [18], an open-source platform for Kubernetes management, providing integrated tools for running containerized applications. Complete code for the web service REST API and GUI is available on Github [39, 40]. For deploying the web service using Kubernetes, Docker containers were used to build an independent service for each receptor. Similarly a separate container was used for the MariaDB database that keeps the docking scores of all the docked ligands. A separate container was also build for the webservice GUI. A bash script [41] was written to deploy all the Docker containers. The bash script applies all kubernetes yaml deployment descriptors that launch the Docker containers. The microservice architecture has many advantages, e.g. independent scaling of services based on usage, cross platform independence and several other inherited benefits of dockerization [42]. All the Docker images are available on Docker Hub [43] with appropriate tags [44–47]. Additionally, users can also create Docker images for new receptors using the Dockerfile available at [48]. A tutorial is available in Additional file 1 explaining how to create and execute Docker images locally. The webpage for the PTPAAS microservice can be accessed at http://ptpaas.service.pharmb.io and the models can also be accessed separately via an OpenAPI interface.

## Results

### Virtual screening evaluation

**Fig. 3:**
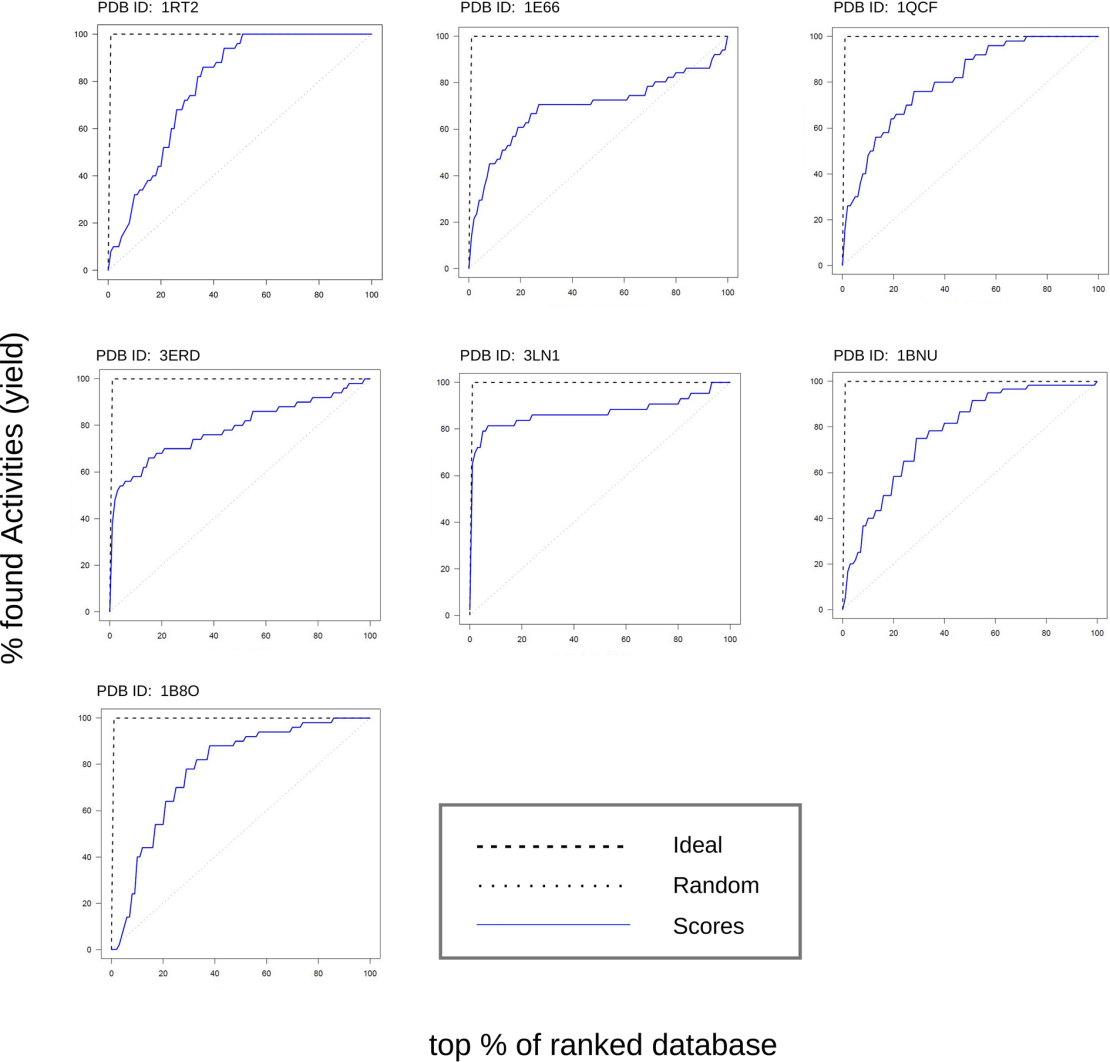
Enrichment curves for Vina docking. In order to verify the virtual screening process, well known inhibitors for each of the 7 receptors were docked using QuickVina and the enrichment factor was computed for the inhibitors docking scores against the docking scores of molecules docked during modelling procedure. Enrichment factor is one of the most common index used for measuring the success of Virtual Screening. Enrichment means where the value lies in the evaluated dataset in comparison to the compared dataset. The higher the enrichment factor, the better the performance of docking in identifying known inhibitors. The black dashed line represents ideal scores, the grey dotted line in the middle represents random scores whereas the blue solid line represents the scores of the inhibitors. For most of the receptors, the results show good or satisfactory enrichment

In order to verify the virtual screening process, we separately docked well-known inhibitors (actives) for each of the 7 receptors using QuickVina and computed the enrichment factor for the inhibitors docking scores against the docking scores of the ligands docked during the modelling procedure. Enrichment factor is one of the most commonly used metrics for measuring the accuracy of virtual screening. Enrichment means where the position of the value is in the evaluated dataset in comparison to the compared dataset. The higher the enrichment factor, the better the performance of docking in identifying known inhibitors. Figure 3 shows the docking enrichment results of QuickVina based CPVS for all the 7 receptors. The black dashed line represents ideal scores, the grey dotted line on the diagonal represents random scores, whereas the blue solid line represents the scores of the known inhibitors. For most of the receptors, the results show good or satisfactory enrichment i.e. well above what would be scores of random ligands and relatively closer to the ideal scores.

**Fig. 4:**
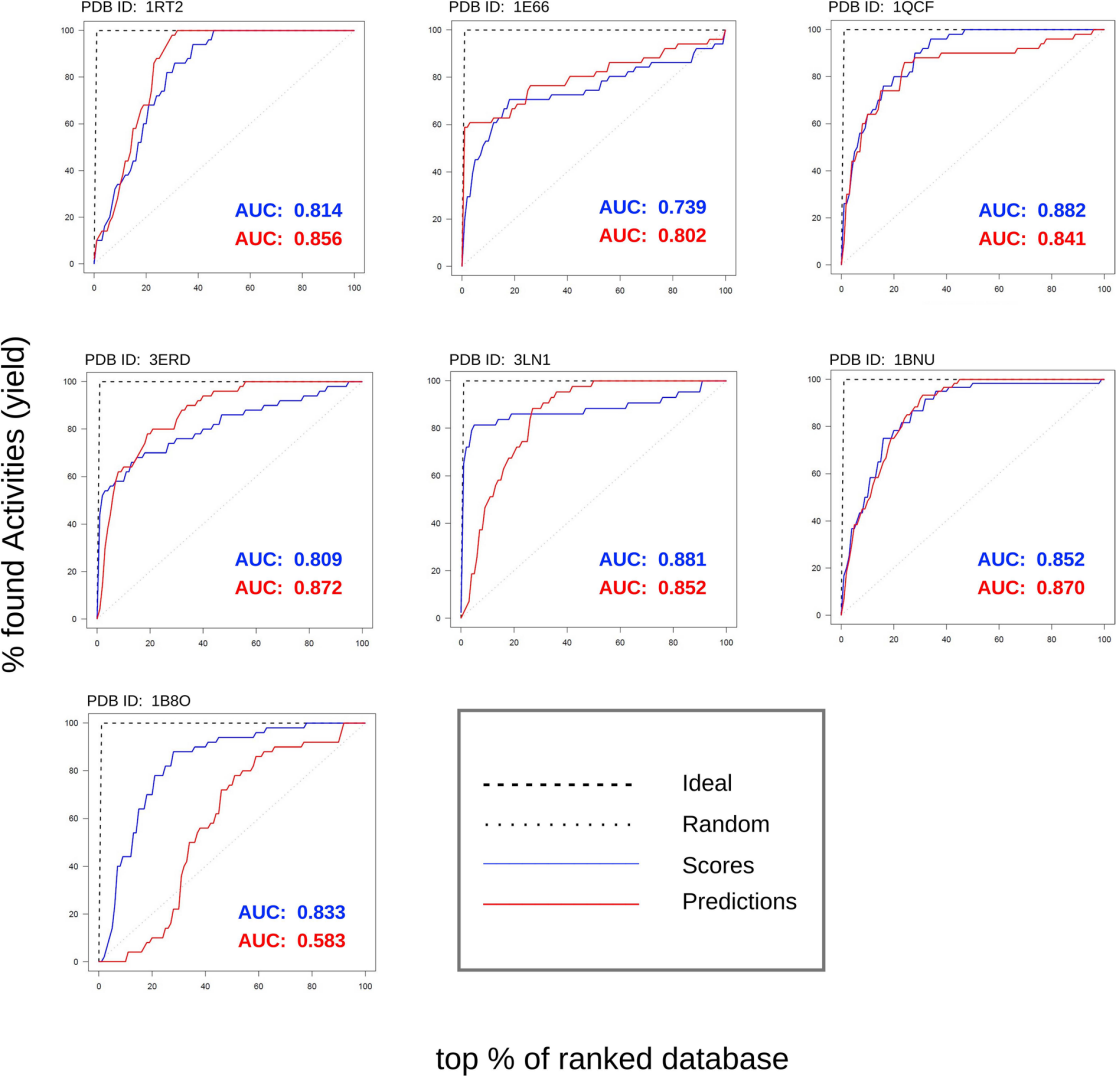
Predicted enrichment vs docking enrichment on the external validation set. The figure presents the comparison of docking enrichment in blue and predicted enrichment in red whereas the grey line in the figure represents random predictions. The comparison was used to evaluate the performance of CPVS models. The docking enrichment was created by comparing docking scores of well known inhibitors and docking scores of an external validation. Similarly the predicted enrichment was created by comparing predicted p-values for well-known inhibitors and the external validation set. AUC was also calculated and reported in the figure for comparison. Overall the CPVS models performed well and predicted enrichment is comparable to docking enrichment, except for receptor with PDB-ID 1B8O, when the predicted enrichment is a little worse than docked enrichment. The reason could be less number of known inhibitors in the top scored molecules, seen in the left bottom corner of the 1B8O graph

We also performed docking enrichment of inhibitors against docking scores of an external validation set which was not seen by the CPVS algorithm during modelling. The docking enrichment can be seen as blue solid line in Fig. 4. The enrichment shows satisfactory results and were used as baseline for evaluating model predictions.

### Model evaluation

The CPVS models were evaluated using multiple methods: (i) by comparing the docking and the predicted enrichment on the external validation set, (ii) by polypharmacology validation i.e. by predicting the activity of known inhibitors for multiple receptors and (iii) by computing the model efficiency.

#### Predicted vs docking enrichment

In Fig. 4, the red line represents the predicted enrichment on the external validation set and the grey line on the diagonal represents random predictions. To generate the predicted enrichment red line, we made predictions using the CPVS models, i.e., the p-values of the inhibitors and the external validation set for being predicted as either a low-scoring or a high-scoring ligand. The p-values were used to compute unary enrichment values by the following formula:

$$\begin{aligned}&\hbox {If} \, ({\textit{P}}_{{\textit{low}}{-}{\textit{scoring}}} > P_{{\textit{high}}{-}{\textit{scoring}}})\\&\quad {\textit{P}}_{{\textit{low}}{-}{\textit{scoring}}} * (1- P_{{\textit{high}}-{\textit{scoring}}})\\&\hbox {else}\\&\quad -{\textit{P}}_{{\textit{high}}-{\textit{scoring}}} * (1- P_{{\textit{low}}-{\textit{scoring}}}) \end{aligned}$$

**Fig. 5:**
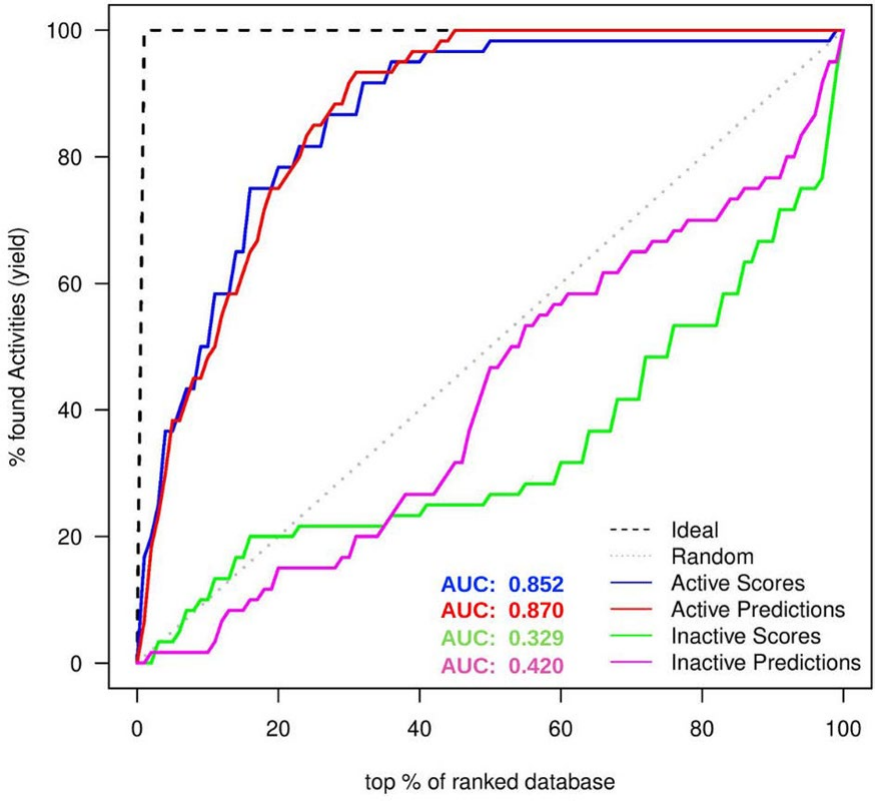
Validating the model for the known in-actives for the receptor 1BNU. The figure presents the comparison of the docking enrichment in green and the predicted enrichment in magenta for the known in-active compounds. The comparison was used to validate the performance of the 1BNU receptor model for the known in-active compounds. The docking enrichment was created by comparing the docking scores of the known in-actives and the docking scores of the external validation set. Similarly the predicted enrichment was created by comparing the predicted p-values for the known in-actives and the p-values for the external validation set. AUC was also calculated and reported in the figure for comparison. Overall, the 1BNU model performed well and the predicted enrichment was comparable to the docking enrichment. The green line for the docking enrichment, which was below the random grey line, also confirms the validity of the virtual screening evaluation

These values were used to create predicted enrichment of known inhibitors against the external validation set. In comparing the predicted enrichment (red solid line) to the docking enrichment (blue solid line), the results were satisfactory for the most of the receptors except for PDB-ID 1B8O. Area under the enrichment curves (AUC) was also calculated and reported in Fig. 4 for comparison.

The number of the known inhibitors found in the top 10% and 20% of the docked molecules and the predicted ligands were also computed and presented in Table 3. The average number of the known inhibitors, for all the receptors, found in the top 20% of the predicted ligands was 63% whereas it was 74% for the docked molecules. In the top 10% of the predicted ligands, the average number of known inhibitors found were 46% whereas in the top 10% of the docked molecules, it was 55%. Again, the receptor with PDB-ID 1B8O was an exception where only 11% of the inhibitors were found in the top 20% of the predicted ligands and none in the top 10%. Inspection of the PDB file for 1B8O did not reveal any obvious explanations for this. The docking works better for some receptors than others and in the case of 1B8O, not many inhibitors were found in the top most scoring ligands (see Fig. 4). This could be one reason of under-performing predicted enrichment for 1B8O.

**Table 3:** The table represents the model efficiency of predictions on the complete modelling set (from which training set was taken) and the external validation set

PDB entry	Eff on modelling set (%)	Eff on ext. val. set (%)	Inhibitors in top 10 (%) predicted ligands	Inhibitors in top 10 (%) docked molecules	Inhibitors in top 20 (%) predicted ligands	Inhibitors in top 20 (%) docked molecules
1RT2	93	97	32	31	68	68
1E66	93	94	60	52	67	70
1QCF	86	93	65	65	73	79
3ERD	93	92	65	58	78	69
3LN1	98	98	50	82	68	86
1BNU	87	87	47	55	75	78
1B8O	94	94	0	43	11	73
Average	92	94	46	55	63	74

The last four columns represents the predicted and the docking enrichment factor for inhibitors, i.e., the percent inhibitors found in the top 10% and 20% of the database search

The methodology was also tested for known in-actives against the external validation set and the results are shown in Fig. 5. The green line represents the docking enrichment of the known in-actives of the 1BNU receptor against the external validation set and the magenta line represents the predicted enrichment of the known in-actives of the 1BNU receptor against the predictions of the external validation set. AUC was also computed and shown in Fig. 5 for comparison. The result is satisfactory, with $\sim$82% of the green line being below the random line. Similarly, the predicted enrichment for the known in-actives (magenta) shows encouraging results as $\sim$98% of it appears below the random line and also near to the docking enrichment green line.

#### Polypharmacology validation

Polypharmacology validation means testing the inhibition of the compounds for multiple targets or disease pathways. A total of 9 compounds were selected from CHEMBL [8] that have a reasonable level of activity for two receptors as given in Table 4. The results were quite good for 4 out of the 9 compounds that were correctly predicted as actives for both of the receptors and only one of the compound was predicted incorrectly as an inactive. In none of the examples, both the compounds were predicted incorrectly as an inactive.

**Table 4:** The table represents the predicted activity of known inhibitors for two compounds

CHEMBL ID	Receptor 1	Receptor 2	Prediction receptor 1	Prediction receptor 2
CHEMBL118	3LN1	1BNU	Active	Active
CHEMBL122708	3ERD	1BNU	Active	Unknown
CHEMBL15841	3ERD	1BNU	active	Inactive
CHEMBL165	3ERD	1BNU	Active	Active
CHEMBL1782957	3ERD	1BNU	Active	Active
CHEMBL1782958	3ERD	1BNU	Active	Unknown
CHEMBL255863	1QCF	1BNU	Unknown	Active
CHEMBL4075710	3ERD	1BNU	Active	Active
CHEMBL66879	3ERD	1BNU	Active	Unknown

Following is the list of 9 compounds with reasonable amount of affinity for a couple of targets to perform polypharmacology validation

#### Efficiency

The models were also evaluated through the measure of efficiency. As mentioned before, the predictions from conformal prediction based classification could be either {0}, {1}, {0, 1} or Ø. Efficiency means the percentage of ligands predicted as low-scoring or high-scoring, i.e., *single predictions* out of the predictions on the complete dataset. Table 3 presents the efficiency of each of the 7 models that are used for predicting the target profiles. All the models created had an efficiency of 80 or higher as intended for both the modelling set and the external validation set. Further details about model efficiency and accuracy can be found in the CPVS paper [37].

## Discussion

Target profiles are utilized to understand the off-target effects of drugs in early stage of drug development. In this work, we present a new way to build prediction based target profiles. We build conformal prediction based machine learning models using the docking scores produced by QuickVina. The process was validated through virtual screening and model evaluation and overall recorded comparable results. Hence, the main finding is that building efficient models for predicting the target profiles are possible through docking scores.

Although previous studies with predictions of ligand-target binding using the docking scores are available, a tool or a web service for predicting target profiles based on docking scores is unavailable to the best of our knowledge; the available web services make use of interaction values from databases. Our work opens up a new direction of using docking scores for predicting target profiles and it would be interesting to compare the two approaches in the future and investigate hybrid system.

The PTPAAS system can be instantiated on other infrastructures such as public cloud providers or on-prem infrastructures (e.g. a company intranet), our deployment at http://ptpaas.service.pharmb.io should be seen as a reference instance. The system has been designed with extensibility in mind, and new models can be deployed as micro services using Docker containers. Such new services (comprising models for new receptors) can be deployed in a similar way as shown for the reference instance on Kubernetes (code and instructions available on [41]). In Additional file 1 we show how users can build models using our previous method [37] and then use the models to create service for a new receptor. Instructions are provided to deploy and add the Docker container for a new receptor to the service [39].

Openness and accessibility are important in science, and hence we switched from OEDocking used in the original CPVS method to QuickVina for docking in this study. The move to QuickVina was quite simple and suggests that the proposed methodology can be used with different docking methods with ease. However, QuickVina is slower and thus restricted us to build limited number of models especially with large datasets. In the future, we would like to add more receptor models, and we encourage the community to contribute to this goal.

## Conclusion

In this paper we present a new methodology for building predicted target profiles using conformal prediction and docking scores from virtual screening. The method was validated through docking of well known inhibitors for each of the 7 receptors. Virtual screening enrichment graphs and model efficiency suggests that docking score based predicted target profiles are a new viable option. The method is made available as a web service with the primary objective to provide predicted target profiles whereas molecular docking is also provided to dock ligands of interest.

## Supplementary information

**Additional file 1.** The file contains a step by step tutorial for running the CPVS API on a local system. It also explains the process of preparing new Docker images for new receptors. Secondly, the file contains various compounds used in the study. Thirdly, it includes property distribution of the known actives and inactives for the receptor 1BNU.

## Data Availability

The clean drug-like molecule library used for our benchmarks can be downloaded from ZINC [19] in ready-to-dock SDF format. The Docker containers for each of the receptor microservice are available on Docker Hub with appropriate tags for each of the receptor and can be reached by searching *cpvsapi* on the Docker Hub website [43]. Additionally, users can also create Docker images for new receptors using the Docker file available at [48].

## Abbreviations

AUC: Area under the curve
NCE: Noval chemical entities
QSAR: Qualitative structure activity relationship
SAR: Structure activity relationship
SVM: Support Vector Machines
PTPAAS: Predicting target profile as a service using docking scores
CPVS: Conformal prediction based virtual screening
RMSD: Root mean square deviation
PDB: Protein data bank
SMILES: Simplified molecular input line entry specification

## References

[1] Yıldırım MA, Goh K-I, Cusick ME, Barabási A-L, Vidal M (2007). Drug target network. Nat Biotechnol.

[2] Hopkins AL (2008). Network pharmacology: the next paradigm in drug discovery. Nat Chem Biol.

[3] Peters J-U (2013). Polypharmacology-foe or friend?. J Med Chem.

[4] Ravikumar B, Aittokallio T (2018). Improving the efficacy-safety balance of polypharmacology in multi-target drug discovery. Expert Opin Drug Discov.

[5] Bowes J, Brown AJ, Hamon J, Jarolimek W, Sridhar A, Waldron G, Whitebread S (2012). Reducing safety-related drug attrition: the use of in vitro pharmacological profiling. Nat Rev Drug Discov.

[6] Cereto-Massagué A, Ojeda MJ, Valls C, Mulero M, Pujadas G, Garcia-Vallve S (2015). Tools for in silico target fishing. Methods.

[7] Sydow D, Burggraaff L, Szengel A, van Vlijmen HW, IJzerman AP, van Westen GJ, Volkamer A (2019). Advances and challenges in computational target prediction. J Chemical Inf Model.

[8] Gaulton A, Bellis LJ, Bento AP, Chambers J, Davies M, Hersey A, Light Y, McGlinchey S, Michalovich D, Al-Lazikani B (2011). Chembl: a large-scale bioactivity database for drug discovery. Nucleic Acids Res.

[9] Sun J, Jeliazkova N, Chupakhin V, Golib-Dzib J-F, Engkvist O, Carlsson L, Wegner J, Ceulemans H, Georgiev I, Jeliazkov V (2017). Excape-db: an integrated large scale dataset facilitating big data analysis in chemogenomics. J Cheminf.

[10] Yu H, Chen J, Xu X, Li Y, Zhao H, Fang Y, Li X, Zhou W, Wang W, Wang Y (2012). A systematic prediction of multiple drug-target interactions from chemical, genomic, and pharmacological data. PloS ONE.

[11] Yao Z-J, Dong J, Che Y-J, Zhu M-F, Wen M, Wang N-N, Wang S, Lu A-P, Cao D-S (2016). Targetnet: a web service for predicting potential drug-target interaction profiling via multi-target SAR models. J Comput Aided Mol Des.

[12] Bender A, Scheiber J, Glick M, Davies JW, Azzaoui K, Hamon J, Urban L, Whitebread S, Jenkins JL (2007). Analysis of pharmacology data and the prediction of adverse drug reactions and off-target effects from chemical structure. ChemMedChem Chem Enab Drug Discov.

[13] Wang L, Ma C, Wipf P, Liu H, Su W, Xie X-Q (2013). Targethunter: an in silico target identification tool for predicting therapeutic potential of small organic molecules based on chemogenomic database. AAPS J.

[14] Awale M, Reymond J-L (2017). The polypharmacology browser: a web-based multi-fingerprint target prediction tool using chembl bioactivity data. J Cheminf.

[15] LaBute MX, Zhang X, Lenderman J, Bennion BJ, Wong SE, Lightstone FC (2014). Adverse drug reaction prediction using scores produced by large-scale drug-protein target docking on high-performance computing machines. PloS ONE.

[16] Wallach I, Jaitly N, Lilien R (2010). A structure-based approach for mapping adverse drug reactions to the perturbation of underlying biological pathways. PloS ONE.

[17] Zsoldos Z, Reid D, Simon A, Sadjad SB, Johnson AP (2007). ehits: a new fast, exhaustive flexible ligand docking system. J Mol Graph Modell.

[18] Run Kubernetes everywhere. https://rancher.com/. **[cito:usesMethodIn]** (2019–2020)

[19] Irwin JJ, Sterling T, Mysinger MM, Bolstad ES, Coleman RG (2012). Zinc: a free tool to discover chemistry for biology. J Chem Inform Model.

[20] Faulon J-L, Visco DP, Pophale RS (2003). The signature molecular descriptor. 1. Using extended valence sequences in GSAR and GSPR studies. J Chem Inf Comput Sci.

[21] Capuccini M, Spark cheminformatics utils. https://github.com/mcapuccini/spark-cheminformatics. **[cito:usesMethodIn]** (2015–2020)

[22] Alvarsson J, Eklund M, Andersson C, Carlsson L, Spjuth O, Wikberg JE (2014). Benchmarking study of parameter variation when using signature fingerprints together with support vector machines. J Chem Inf Model.

[23] Cortes C, Vapnik V (1995). Support vector networks. Mach Learn.

[24] Trott O, Olson AJ (2010). Autodock vina: improving the speed and accuracy of docking with a new scoring function, efficient optimization, and multithreading. J Comput Chem.

[25] Alhossary A, Handoko SD, Mu Y, Kwoh C-K (2015). Fast, accurate, and reliable molecular docking with quickvina 2. Bioinformatics.

[26] Jones G, Willett P, Glen RC, Leach AR, Taylor R (1997). Development and validation of a genetic algorithm for flexible docking. J Mol Biol.

[27] Kellenberger E, Muller P, Schalon C, Bret G, Foata N, Rognan D (2006). sc-pdb: an annotated database of druggable binding sites from the protein data bank. J Chem Inf Model.

[28] O’Boyle NM, Banck M, James CA, Morley C, Vandermeersch T, Hutchison GR (2011). Open babel : an open chemical toolbox. J Cheminf.

[29] Andersson CD, Thysell E, Lindström A, Bylesjö M, Raubacher F, Linusson A (2007). A multivariate approach to investigate docking parameters’ effects on docking performance. J Chem Inf Model.

[30] Wishart DS, Knox C, Guo AC, Cheng D, Shrivastava S, Tzur D, Gautam B, Hassanali M (2007) Drugbank: a knowledgebase for drugs, drug actions and drug targets. Nucleic acids research 36(suppl$$\_$$1):901–906 **[cito:citesAsDataSource]** 10.1093/nar/gkm958 PMC2238889 18048412

[31] Vovk V, Gammerman A, Shafer G (2005). Algorithmic learning in a random world.

[32] Norinder U, Carlsson L, Boyer S, Eklund M (2014). Introducing conformal prediction in predictive modeling. a transparent and flexible alternative to applicability domain determination. J Chem Inf Model.

[33] Vovk V, Fedorova V, Nouretdinov I, Gammerman A (2016) Criteria of efficiency for conformal prediction. In: Symposium on conformal and probabilistic prediction with applications. Springer, pp 23–39. **[cito:citesAsAuthority]**

[34] Norinder U, Boyer S (2017). Binary classification of imbalanced datasets using conformal prediction. J Mol Graph Modell.

[35] Sahigara F, Mansouri K, Ballabio D, Mauri A, Consonni V, Todeschini R (2012). Comparison of different approaches to define the applicability domain of GSAR models. Molecules.

[36] Gammerman A, Vovk V (2007). Hedging predictions in machine learning. Comput J.

[37] Ahmed L, Georgiev V, Capuccini M, Toor S, Schaal W, Laure E, Spjuth O (2018). Efficient iterative virtual screening with apache spark and conformal prediction. J Cheminf.

[38] Drobi S (2012). Play2: a new era of web application development. IEEE Internet Comput.

[39] Ahmed L. Rest API for CPVS. https://github.com/laeeq80/cpvsAPI (2019–2020)

[40] Ahmed L. User Interface for CPVSAPI. https://github.com/laeeq80/cpvs-ui (2019–2020)

[41] Larsson A. Kubernetes deployment of ptdpaas. https://github.com/pharmbio/dpaas. **[cito:usesMethodIn]** (2019–2020)

[42] Merkel D (2014). Docker: lightweight Linux containers for consistent development and deployment. Linux J.

[43] Docker Hub. https://hub.docker.com/. **[cito:usesMethodIn]** (2014–2020)

[44] Ahmed L. Docker Image for CPVS API on Docker Hub. https://hub.docker.com/r/laeeq/cpvsapi (2019–2020)

[45] Ahmed L. Docker Image for CPVS UI on Docker Hub. https://hub.docker.com/r/laeeq/cpvs-ui (2019–2020)

[46] Ahmed L. Docker Image for Custom MariaDB on Docker Hub. https://hub.docker.com/r/laeeq/ligandprofiledb (2019–2020)

[47] Ahmed L. Docker Image on Docker Hub to Upload PDBQT file to the web service. https://hub.docker.com/r/laeeq/uploadfile (2019–2020)

[48] Ahmed L. Docker File for CPVSAPI Project. https://github.com/laeeq80/cpvsDocker (2018–2020)

[49] Toor S, Lindberg M, Falman I, Vallin A, Mohill O, Freyhult P, Nilsson L, Agback M, Viklund L, Zazzik H, et al. (2017) Snic science cloud (ssc): a national-scale cloud infrastructure for swedish academia. In: 2017 IEEE 13th international conference on e-Science (e-Science), IEEE, New York, pp 219–227

