# Supplementary material for: Predicting target profiles with confidence as a service using docking scores
Source: J Cheminform. 2020 Oct 15;12:62. doi: 10.1186/s13321-020-00464-1 (PMC7566026; doi:10.1186/s13321-020-00464-1)
Supplement: Supplementary file 1 — Additional file 1. The file contains a step by step tutorial for running the CPVS API on a local system. It also explains the process of preparing new Docker images for new receptors. Secondly, the file contains various compounds used in the study. Thirdly, it includes property distribution of the known actives and inactives for the receptor 1BNU. [file 13321_2020_464_MOESM1_ESM.pdf]

Additional file 1

Step by step process for running CPVSAPI on a local system

Note: For this material, we assume that Docker and Git are already installed on your local system. If you want to prepare the Docker images yourself, then first perform section 2 and then start from section 1.2. Section 3 contains some extra docker commands which can be useful if you want to test both ready made and custom build docker images.

0. Test your Docker installation

```
docker run hello-world
```

You would need to use **sudo** in front of all the docker commands if post docker installation was not followed. Details are available here https://docs.docker.com/install/linux/linux-postinstall/

1. Executing the Docker images

1.1 Pulling readymade Docker images

1.1.1 To pull MariaDB database

```
docker pull laeeq/ligandprofiledb:0.0.3
```

1.1.2 To pull CPVSAPI for 1QCF

```
docker pull laeeq/cpvsapi:1QCF-0.0.1
```

1.2 Starting MariaDB Container from Image in background (using detach)

```
docker run --detach --name test-mariadb -d laeeq/ligandprofiledb:0.0.3
```

1.3 Checking container status

```
docker ps
```

1.4 Want to check what happened

```
docker logs test-mariadb
```

1.5 Finding IP address of container

```
docker inspect test-mariadb | grep IPAddress
```

Now we know the IP Address where the database is running, so we would be able to connect it. In our case, it was 172.17.0.2.

Note: Make sure to write it down, you will need it in step 1.11.

1.6 Logging into MariaDB container and start a bash environment

```
docker exec -it test-mariadb bash
```

1.7 Logging into MariaDB in the Docker container by using the following command

```
mysql -uroot -pmariadb_root
```

1.8 Allowing other Docker containers to access MariaDB

```
GRANT ALL PRIVILEGES ON *.* TO 'root'@'172.17.0.%' IDENTIFIED BY
'mariadb_root' WITH GRANT OPTION;
```

1.9 Exit from MariaDB

```
exit
```

1.10 Exit from MariaDB container

```
exit
```

1.11 Running the CPVSAPI Docker container for 1QCF receptor and linking to MariaDB container

You must use the correct MARIADB_IP you found in the section 1.5, otherwise the cpvsapi docker container won't be able to connect the MariaDB database.

```
docker run --detach --name test-cpvs -e MARIADB_IP='172.17.0.2' -e
MARIADB_PASSWORD='mariadb_root' -e RECEPTOR_NAME='HCK Tyrosine kinase' -e
RECEPTOR_PDBCODE='1QCF' --link test-mariadb:mariadb -p 9000:9000
laeeq/cpvsapi:1QCF-0.0.1
```

So what have we done here? We executed a new container test-cpvs and using `--link` we linked it to the test-mariadb docker container which was already running. `-p 9000:9000` is used to publish test-cpvs port to localhost.

1.12 Trying out the cpvs rest api for one receptor i.e. 1QCF

Open any web browser and access the 9000 port of the running test-cpvs Docker container we published to the local machine. Try the following in any web browser.

```
http://localhost:9000
```

If everything works, you should see a swagger rest ui for cpvs. There are three end points of the CPVS API that can be tested i.e. predictions, pvalues and docking. Click anyone by ***try it out*** by giving compound in SMILES format. A sample SMILE is given below:

```
CC(C)c1ccc(Nc2nccc(n2)c3cnn4ncccc34)cc1
```

2. Preparing Docker images

### Java 8, Maven and sbt 1.1.6 must be installed on local for section 2 and JAVA_HOME must be set ###

2.1 Installing Project Dependencies

Clone spark-cheminformatics utilities for tools like signature generation

```
git clone https://github.com/mcapuccini/spark-cheminformatics.git
```

Enter the newly cloned directory. There are two projects **parsers and sg**. Enter **both** of them and run the following maven command to install each one of them as local dependencies.

```
mvn clean install -DskipTests
```

Clone spark-cpvs-vina project

```
git clone https://github.com/laeeq80/spark-cpvs-vina.git
```

Enter the project “**vs”** inside spark-cpvs-vina and run the command

```
mvn clean install -DskipTests
```

2.2 Building MariaDB image and copying the database that contains model

We need a **Docker container for MariaDB** that stores the models created using cpvs project.

2.2.1 Clone the ligandprofiledb repo

Use git clone to clone the repo https://github.com/laeeq80/ligandProfiledb to create MariaDB Docker image including the database with 1QCF model.

```
git clone https://github.com/laeeq80/ligandProfiledb.git
```

2.2.2 Build the ligandprofiledb image

Use the following command to create the Docker image at CLI from inside the cloned directory. The Dockerfile also includes the database copying step.

Enter the ligandprofiledb directory and run the following command

```
docker build . -t laeeq/ligandprofiledb:0.0.3
```

!! Step 6/8 in the Dockerfile can take upto 5 minutes. **Grab a coffee.**

2.3 Building cpvsapi Docker image for one of the receptor i.e. with PDB ID "1QCF". The pdbqt for the 1QCF is already available in the cpvsapi repo.

2.3.1 Clone the cpvsapi repo

Get out of the ligandprofiledb directory and run the following command.

```
git clone https://github.com/laeeq80/cpvsAPI.git
```

The pdbqt for the 1QCF and other resources are already available in the cpvsapi repo resources folder.

2.3.2 Creating package

Enter the cpvsAPI and run the following command

```
sbt dist
```

The above command will create cpvsapi-1.0.zip file in the **cpvsAPI/target/universal/** directory that is needed while building cpvsapi Docker image in step 2.3.4. In addition, openbabel-2.4.1.tar.gz will also be required that the application uses to convert files into different formats. Openbabel can be downloaded online using

```
wget  https://sourceforge.net/projects/openbabel/files/openbabel/2.4.1/openbabel-2.4.1.tar.gz
```

If wget is not available on your system, you can use a web browser to get it.

2.3.3 Clone cpvsDocker Dockerfile

```
git clone https://github.com/laeeq80/cpvsDocker.git
```

2.3.4 Build Docker image for cpvsapi

Copy cpvsapi-1.0.zip and openbabel-2.4.1.tar.gz inside the cpvsDocker directory and run the following command from cpvsDocker directory.

```
docker build . -t laeeq/cpvsapi:1QCF-0.0.1
```

This will take around **15 to 20 minutes**. **Grab another coffee. [:D]**

Goto Step 1.2 to test the newly created Docker images.

3. Some helpful commands

3.1 List all images

```
docker image ls
```

3.2 Deleting a docker image

```
docker rmi imageID
```

3.3 List running docker containers

```
docker ps
```

3.4 List already stopped but unremoved containers

```
docker ps -a
```

3.5 Stopping and removing a running docker container

```
docker stop containerName
```

```
docker rm containerName
```

E.g.

```
docker stop test-cpvs
```

List of Known Inhibitors in SMILES format

1. 1RT2

COC(=N[C@H](C(c1ccccc1)c2ccccc2)C(=N[C@@H](c3ccc(s3)[C@H](CO)N(CCC(C)(C)C)S(=O)(=O)c4cc(F)c(CO)c(F)c4)C(F)(F)F)O)O
COC(=O)N[C@@H](C(c1ccccc1)c2ccccc2)C(=O)NCC(F)(F)CC[C@@H](CO)N(CC3CC(F)(F)C3)S(=O)(=O)c4ccc5ncsc5c4
COC(=N[C@@H](C(c1ccccc1)c2ccccc2)C(=NCc3ccc(s3)C(CO)N(CCC(C)C)S(=O)(=O)c4ccc(CO)cc4)O)O
CCC(N=C(O)[C@@H](N=C(O)OC)C(c1ccccc1)c2ccccc2)c3ccc(s3)C(CO)N(CCC(C)C)S(=O)(=O)c4ccc(CO)cc4
COC(=O)N[C@@H](C(c1ccccc1)c2ccccc2)C(=O)NCC(F)(F)CC[C@H](N(CC3CC(F)(F)C3)S(=O)(=O)c4ccc5ncsc5c4)C(=O)N
COC(=N[C@H](C(c1ccccc1)c2ccccc2)C(=N[C@@H](c3ccc(s3)[C@H](CO)N(CCC(C)(C)C)S(=O)(=O)c4ccc(cc4)c5oncc5)C(F)(F)F)O)O
CC(C)CN(C(CO)c1ccc(CN=C(O)C(N=C(O)c2ccccc2)C(c3ccccc3)c4cccc5ncccc45)s1)S(=O)(=O)c6ccc7ncsc7c6
COC(=O)N[C@@H](C(c1ccccc1)c2ccccc2)C(=O)NCC(F)(F)CC[C@@H](CO)N(Cc3cn[nH]c3)S(=O)(=O)c4ccc5ncsc5c4
COC(=N[C@H](C(c1ccccc1)c2ccccc2)C(=NCc3ccc(s3)[C@H](CO)N(CC4CC(F)(F)C4)S(=O)(=O)c5ccc6CCOCc6c5)O)O
COC(=N[C@H](C(c1ccccc1)c2ccccc2)C(=NCc3ccc(s3)[C@H](CO)N(CC4CC(F)(F)C4)S(=O)(=O)c5ccc(N)cc5)O)O
COC(=N[C@H](C(c1ccccc1)c2ccccc2)C(=N[C@@H](c3ccc(s3)[C@H](CO)N(CCC(C)(C)C)S(=O)(=O)c4ccc(N)cc4F)C(F)(F)F)O)O
CC(C)CN(C(CO)c1ccc(CN=C(O)C(N=C(O)c2ccccc2)C(c3ccccc3)c4ccnc5ccccc45)s1)S(=O)(=O)c6ccc7ncsc7c6
COC(=N[C@H](C(c1ccccc1)c2ccccc2)C(=N[C@@H](c3ccc(s3)[C@H](CO)N(CCC(C)(C)C)S(=O)(=O)c4ccc(N)cc4Cl)C(F)(F)F)O)O
COC(=N[C@H](C(c1ccccc1)c2ccccc2)C(=N[C@@H](c3ccc(s3)[C@H](CO)N(CCC(C)(C)C)S(=O)(=O)c4ccc(N)cn4)C(F)(F)F)O)O
COC(=N[C@H](C(c1ccccc1)c2ccccc2)C(=N[C@@H](c3ccc(s3)[C@H](CO)N(CCC(C)(C)C)S(=O)(=O)c4ccc5[nH]ccc5c4)C(F)(F)F)O)O

COC(=N[C@H](C(c1ccccc1)c2ccccc2)C(=N[C@@H](c3ccc(s3)[C@H](CO)N(CCC(C)(C)C)S(=O)(=O)c4ccc5ocnc5c4)C(F)(F)F)O)O
COC(=N[C@H](C(c1ccccc1)c2ccccc2)C(=N[C@@H](c3ccc(s3)[C@H](CO)N(CCC(C)(C)C)S(=O)(=O)c4ccn5ncnc5c4)C(F)(F)F)O)O
COC(=N[C@H](C(c1ccccc1)c2ccccc2)C(=N[C@@H](c3ccc(s3)[C@H](CO)N(CCC(C)(C)C)S(=O)(=O)c4ccc5ncnn5c4)C(F)(F)F)O)O
CCCCCN([C@H](CO)CCC(F)(F)CNC(=O)[C@@H](NC(=O)OC)C(c1ccccc1)c2ccccc2)S(=O)(=O)c3ccc(CO)cc3
COC(=N[C@H](C(c1ccccc1)c2ccccc2)C(=NCc3ccc(s3)[C@H](CO)N(CC(C)C)S(=O)(=O)c4ccc(N)cc4)O)O
COC(=N[C@H](C(c1ccccc1)c2ccccc2)C(=N[C@@H](c3ccc(s3)[C@H](CO)N(CC(C)C)S(=O)(=O)c4ccc(N)cc4)C(F)(F)F)O)O
COC(=N[C@H](C(c1ccccc1)c2ccccc2)C(=N[C@@H](c3ccc(s3)[C@H](CO)N(CCC(C)(C)C)S(=O)(=O)c4ccc(N)cc4)C(F)(F)F)O)O
COC(=N[C@H](C(c1ccccc1)c2ccccc2)C(=N[C@@H](c3ccc(s3)[C@H](CO)N(CC4CC(F)(F)C4)S(=O)(=O)c5ccc(N)cc5)C(F)(F)F)O)O
COC(=N[C@H](C(c1ccccc1)c2ccccc2)C(=N[C@@H](c3ccc(s3)[C@H](CO)N(CCC(C)(C)C)S(=O)(=O)c4ccc5nc[nH]c5c4)C(F)(F)F)O)O
COC(=N[C@H](C(c1ccccc1)c2ccccc2)C(=N[C@@H](c3ccc(s3)[C@H](CO)N(CCC(C)(C)C)S(=O)(=O)c4ccc5nn[nH]c5c4)C(F)(F)F)O)O
COC(=N[C@H](C(c1ccccc1)c2ccccc2)C(=N[C@@H](c3ccc(s3)[C@H](CO)N(CCC(C)(C)C)S(=O)(=O)c4ccc5ncsc5c4)C(F)(F)F)O)O
CC(C)CN(C(CO)c1ccc(CN=C(O)C(N=C(O)c2ccccc2)C(c3ccccc3)c4cccc5cccnc45)s1)S(=O)(=O)c6ccc7ncsc7c6
COC(=N[C@H](C(c1ccccc1)c2ccccc2)C(=N[C@@H](c3ccc(s3)[C@H](CO)N(CCC(F)(F)F)S(=O)(=O)c4ccc5ncsc5c4)C(F)(F)F)O)O
COC(=N[C@H](C(c1ccccc1)c2ccccc2)C(=NCc3ccc(s3)[C@H](CO)N(Cc4ccnc(OC)c4)S(=O)(=O)c5ccc6scnc6c5)O)O
COC(=N[C@H](C(c1ccccc1)c2ccccc2)C(=N[C@@H](c3ccc(s3)[C@H](CO)N(CCC(C)(C)C)S(=O)(=O)c4ccc5[nH]ncc5c4)C(F)(F)F)O)O
COC(=N[C@H](C(c1ccccc1)c2ccccc2)C(=N[C@@H](c3ccc(s3)[C@H](CO)N(CCC(C)(C)C)S(=O)(=O)c4ccc5ncccc5c4)C(F)(F)F)O)O
COC(=N[C@H](C(c1ccccc1)c2ccccc2)C(=N[C@@H](c3ccc(s3)[C@H](CO)N(C(C)C)S(=O)(=O)c4ccc(CO)cc4)C(F)(F)F)O)O
COC(=N[C@H](C(c1ccccc1)c2ccccc2)C(=N[C@@H](c3ccc(s3)[C@H](CO)N(CCCC(F)(F)F)S(=O)(=O)c4ccc5ncsc5c4)C(F)(F)F)O)O
COC(=O)N[C@@H](C(c1ccccc1)c2ccccc2)C(=O)NCC(F)(F)CC[C@@H](CO)N(C3CCC(F)(F)CC3)S(=O)(=O)c4ccc5ncsc5c4
COC(=N[C@H](C(c1ccccc1)c2ccccc2)C(=N[C@@H](c3ccc(s3)[C@H](CO)N(CCC(F)(F)F)S(=O)(=O)c4ccc(N)cc4)C(F)(F)F)O)O
COC(=N[C@H](C(c1ccccc1)c2ccccc2)C(=N[C@@H](c3ccc(s3)[C@H](CO)N(CCC(C)(C)C)S(=O)(=O)c4ccc(N)c(F)c4)C(F)(F)F)O)O
CCCCN([C@@H](CO)c1ccc(s1)[C@H](N=C(O)[C@H](N=C(O)OC)C(c2ccccc2)c3ccccc3)C(F)(F)F)S(=O)(=O)c4ccc(N)cc4
COC(=O)N[C@@H](C(c1ccccc1)c2ccccc2)C(=O)NCC(F)CC[C@@H](CO)N(CCC(C)C)S(=O)(=O)c3ccc(N)cc3
COC(=N[C@H](C(c1ccccc1)c2ccccc2)C(=N[C@@H](c3ccc(s3)[C@H](CO)N(CCC4CC4)S(=O)(=O)c5ccc(N)cc5)C(F)(F)F)O)O
COC(=N[C@H](C(c1ccccc1)c2ccccc2)C(=N[C@@H](c3ccc(s3)[C@H](CO)N(CCc4ccccc4)S(=O)(=O)c5ccc(N)cc5)C(F)(F)F)O)O
COC(=N[C@@H](C(c1ccccc1)c2ccccc2)C(=NC(C)c3ccc(s3)C(CO)N(CCC(C)C)S(=O)(=O)c4ccc(CO)cc4)O)O
COC(=N[C@H](C(c1ccccc1)c2ccccc2)C(=N[C@@H](c3ccc(s3)[C@H](CO)N(CCC(C)(C)C)S(=O)(=O)c4ccc(CO)cc4)C(F)(F)F)O)O
COC(=N[C@H](C(c1ccccc1)c2ccccc2)C(=N[C@@H](c3ccc(s3)[C@H](CO)N(CCC(C)(C)C)S(=O)(=O)c4ccc5cc[nH]c5c4)C(F)(F)F)O)O
COC(=N[C@H](C(c1ccccc1)c2ccccc2)C(=NCc3ccc(s3)[C@H](CO)N(Cc4cn[nH]c4)S(=O)(=O)c5ccc6scnc6c5)O)O
COC(=N[C@H](C(c1ccccc1)c2ccccc2)C(=NCc3ccc(s3)[C@H](CO)N(CC4CC(F)(F)C4)S(=O)(=O)c5ccc6ccnc(O)c6c5)O)O
COC(=N[C@H](C(c1ccccc1)c2ccccc2)C(=N[C@@H](c3ccc(s3)[C@H](CO)N(CCC(C)(C)C)S(=O)(=O)c4ccc5snnc5c4)C(F)(F)F)O)O
CC(C)CN(C(CO)c1ccc(CN=C(O)C(N=C(O)c2ccccc2)C(c3ccccc3)c4c[nH]c5ncccc45)s1)S(=O)(=O)c6ccc7ncsc7c6
COC(=N[C@H](C(c1ccccc1)c2ccccc2)C(=N[C@@H](c3ccc(s3)[C@H](CO)N(CCC(C)C)S(=O)(=O)c4ccc(N)cc4)C(F)(F)F)O)O
COC(=N[C@H](C(c1ccccc1)c2ccccc2)C(=N[C@@H](c3ccc(s3)[C@H](CO)N(CCC(C)(C)C)S(=O)(=O)c4ccc5c[nH]nc5c4)C(F)(F)F)O)O
COC(=N[C@H](C(c1ccccc1)c2ccccc2)C(=NCc3ccc(s3)[C@H](CO)N(CC4CC(F)(F)C4)S(=O)(=O)c5cccc(Cc6ccccn6)c5)O)O

2. 1E66

Nc1c2CCCCCc2nc3cccc(Cl)c13
CC(=O)C(Cc1ccc(O)cc1)C(=O)NCCCNc2c3CCCCc3nc4ccccc24
C\C=C\1/[C@@H]2CC3=C(C=CC(=O)N3)[C@]1(CC(=C2)C)\N=C\c4ccc(cc4)C#N

Clc1ccc2c(Cl)c3CCCCc3nc2c1
CN(C)C(=O)Oc1cccc(c1)[N+](C)(C)C
Cc1cccc(Nc2ccccc2C(=O)NCCCCCCCCNc3c4CCCCc4nc5ccccc35)c1C
CC(C)C1=N[C@H]2CC[C@]34C[C@@]35[C@@H](CC[C@H]4[C@]2(C)CN1)[C@]6(C)C[C@@H](O)[C@H]([C@H](C)N(C)C)[C@@]6(C)CC5=O
O=C1NC2=C(C=C1)[C@H](CCC2)NCCCCCCCCCCN[C@H]3CCCC4=C3C=CC(=O)N4
Cc1cccc(Nc2ccccc2C(=O)NCCCCCCNc3c4CCCCc4nc5cc(Cl)ccc35)c1C
Cc1cccc(Nc2ccccc2C(=O)NCCC(=O)NCCCCNc3c4CCCCc4nc5ccccc35)c1C
COc1ccc(\C=N\[C@@]23CC(=C[C@@H](CC4=C2C=CC(=O)N4)/C/3=C\C)C)c(OC)c1
Cc1nc2cccc(F)c2c(N)c1CSCc3cccc(c3)C(=O)C(F)(F)F
COc1ccc2C=[N+](CCCCCCCCN3C(=O)c4ccccc4C3=O)CC[C@@]56C=C[C@H](O)C[C@@H]5Oc1c26
Cc1cccc(Nc2ccccc2C(=O)NCCCCCCCCCNc3c4CCCCc4nc5ccccc35)c1C
Cc1cccc(Nc2ccccc2C(=O)NCCCCCCNc3c4CCCCc4nc5ccccc35)c1C
Cc1cccc(Nc2ccccc2C(=O)NCC(=O)NCCCNc3c4CCCCc4nc5cc(Cl)ccc35)c1C
Cc1cccc(Nc2ccccc2C(=O)NCCC(=O)NCCCCCCNc3c4CCCCc4nc5cc(Cl)ccc35)c1C
CC(=O)CC(=O)NCCCCCCNc1c2CCCCc2nc3ccccc13
Cc1cccc(Nc2ccccc2C(=O)NCC(=O)NCCCCCCCNc3c4CCCCc4nc5cc(Cl)ccc35)c1C
Cc1cccc(Nc2ccccc2C(=O)NCCCCCC(=O)NCCCCNc3c4CCCCc4nc5cc(Cl)ccc35)c1C
CNC(=O)Oc1ccc2N(C)[C@H]3N(C)CC[C@@]3(C)c2c1.OC(=O)c4ccccc4O
C(CCCNCc1oc2ccccc2c1)CCCNc3c4CCCCc4nc5ccccc35
Cc1cccc(Nc2ccccc2C(=O)NCC(=O)NCCCCCCNc3c4CCCCc4nc5cc(Cl)ccc35)c1C
C\C=C\1/[C@@H]2CC3=C(C=CC(=O)N3)[C@]1(CC(=C2)C)\N=C\C=C\c4ccccc4
COc1cc(OC)cc(\C=N\[C@@]23CC(=C[C@@H](CC4=C2C=CC(=O)N4)/C/3=C\C)C)c1
C\C=C\1/[C@@H]2CC3=C(C=CC(=O)N3)[C@]1(CC(=C2)C)\N=C\C=C\c4ccc(cc4)N(C)C
Cc1cccc(Nc2ccccc2C(=O)NCCCCCCCCCCNc3c4CCCCc4nc5cc(Cl)ccc35)c1C
CC[C@H](C)C1=N[C@H]2CC[C@]34C[C@@]35[C@@H](CC[C@H]4[C@]2(C)CN1)[C@]6(C)C[C@@H](O)[C@H]([C@H](C)N(C)C)[C@@]6(C)CC5=O
Cc1cccc(Nc2ccccc2C(=O)NCCCCCCCCNc3c4CCCCc4nc5cc(Cl)ccc35)c1C
Cc1cccc(Nc2ccccc2C(=O)NCCCCCC(=O)NCCCNc3c4CCCCc4nc5cc(Cl)ccc35)c1C
Cc1nc2cccc(Cl)c2c(N)c1COCc3cccc(c3)C(=O)C(F)(F)F
O=C1NC2=C(C=C1)[C@H](CCC2)NCCCCCCCCCCCCN[C@H]3CCCC4=C3C=CC(=O)N4
COc1ccc(\C=N\[C@@]23CC(=C[C@@H](CC4=C2C=CC(=O)N4)/C/3=C\C)C)cc1
Cc1cccc(Nc2ccccc2C(=O)NCCC(=O)NCCCCCCCCNc3c4CCCCc4nc5ccccc35)c1C
Cc1cccc(Nc2ccccc2C(=O)NCCCCCC(=O)NCCCCCCNc3c4CCCCc4nc5cc(Cl)ccc35)c1C
Cc1cccc(Nc2ccccc2C(=O)NCCCC(=O)NCCCNc3c4CCCCc4nc5cc(Cl)ccc35)c1C
COc1cc(cc(OC)c1OC)C(=O)NCCCCCCCCC(=O)NNc2c3CCCCc3cc4ccccc24
Cc1nc2ccccc2c(N)c1COCc3cccc(c3)C(=O)C(F)(F)F
Cc1cccc(Nc2ccccc2C(=O)NCCCCCCCCCCNc3c4CCCCc4nc5ccccc35)c1C
Cc1cccc(Nc2ccccc2C(=O)NCCCCCCCNc3c4CCCCc4nc5ccccc35)c1C
Cc1cccc(Nc2ccccc2C(=O)NCCC(=O)NCCCCCCCNc3c4CCCCc4nc5ccccc35)c1C
Cc1cccc(Nc2ccccc2C(=O)NCCC(=O)NCCCCCCCCNc3c4CCCCc4nc5cc(Cl)ccc35)c1C
Cc1cccc(Nc2ccccc2C(=O)NCCCCCC(=O)NCCCCCCCCNc3c4CCCCc4nc5ccccc35)c1C
Clc1cccc2nc3CCCCCc3c(NCc4cccs4)c12
Cc1cccc(Nc2ccccc2C(=O)NCC(=O)NCCCCCCCCNc3c4CCCCc4nc5ccccc35)c1C

Cc1cccc(Nc2ccccc2C(=O)NCCCCCC(=O)NCCCCCCCNc3c4CCCCc4nc5ccccc35)c1C
Cc1cccc(Nc2ccccc2C(=O)NCC(=O)NCCCCCCCNc3c4CCCCc4nc5ccccc35)c1C
C\C=C\1/[C@@H]2CC3=C(C=CC(=O)N3)[C@]1(CC(=C2)C)\N=C\C(=C\c4ccccc4)\C
Cc1cccc(Nc2ccccc2C(=O)NCCCCCCNc3c4CCCCc4nc5ccccc35)c1C
Cc1nc2cccc(F)c2c(N)c1COCc3cccc(c3)C(=O)C(F)(F)F
CCOc1cc(\C=N\[C@@]23CC(=C[C@@H](CC4=C2C=CC(=O)N4)/C/3=C\C)C)ccc1O

3. 1QCF

Cc1ccc2c(c3ccnc(Nc4ccc5OCCOc5c4)n3)c(nn2n1)c6ccc(cc6)C(F)(F)F
Cc1ccc(s1)n2nc(cc2NC(=O)Nc3ccc(Oc4ccnc5NC(=O)C=Nc45)c6ccccc36)C(C)(C)C
Fc1ccc(cc1)c2ncn(C3CCNCC3)c2c4ccnc(Oc5cccc(F)c5)n4
COCC(=O)Nc1cc(Oc2ccc(NC(=O)Nc3cc(nn3c4ccc(C)cc4)C(C)(C)C)c5ccccc25)ccn1
CN1CCC(CC1)n2cnc(c3ccc(F)cc3)c2c4ccnc(Nc5ccccc5)n4
Nc1ncnc2c1c(nn2[C@@H]3CCCN(C3)C(=O)C=C)c4ccc(Oc5ccccc5)cc4
Cc1ccc(cc1)n2nc(cc2NC(=O)Nc3ccc(Oc4ccnc5NC(=O)Nc45)c6ccccc36)C(C)(C)C
Nc1ncnc2c1c(nn2C3CCCC3)c4ccc(Cl)c(O)c4
Cc1nc(Nc2ncc(s2)C(=O)Nc3c(C)cccc3Cl)cc(n1)N4CCN(CCO)CC4
Fc1ccc(cc1)c2ncn(C3CCNCC3)c2c4ccnc(Oc5ccc(Oc6ccccc6)cc5)n4
Cc1ccc(cc1)n2cnc3cc(Nc4nnc(C)c5ccccc45)ccc23
COc1cc(ccc1N)c2nn(C3CCCC3)c4ncnc(N)c24
Clc1ccc(cc1)C2=C(N3CCc4ccccc34)C(=O)NC2=O
CS(=O)(=O)c1cccc(Nc2nccc(n2)c3sc(nc3c4cccc(NS(=O)(=O)c5c(F)cccc5F)c4)N6CCOCC6)c1
Cc1ccc2cc([nH]c2c1)c3n[nH]c4ccc(NC5CCN(Cc6ccccc6)CC5)cc34
OC(=O)c1cc(NC2=C(C(=O)NC2=O)c3ccc(Cl)cc3)ccc1Cl
Nc1ncnc2c1c(nn2[C@@H]3CC[C@@H](CC3)NC(=O)C=C)c4ccc(Oc5ccccc5)cc4
COc1ccc(cc1)n2nc(cc2NC(=O)Nc3ccc(Oc4ccnc5NC(=O)N(C)c45)c6ccccc36)C(C)(C)C
FC(F)(F)c1ccc(cc1)c2nn3ncccc3c2c4ccnc(Nc5ccc6OCCOc6c5)n4
CC(C)(C)c1cc(NC(=O)Nc2ccc(Oc3ccnc4NC(=O)C=Nc34)c5ccccc25)n(n1)c6ccc(Cl)c(Cl)c6
Fc1ccc(Oc2nccc(n2)c3c(ncn3C4CCNCC4)c5ccc(F)cc5)cc1
FC(F)(F)c1cccc(Nc2nccc(n2)c3cnn4nc(ccc34)c5ccccc5)c1
O=C1Nc2ccc(N\C=C\3/C(=O)Nc4ccccc34)cc2N1
CC(C)n1nc(c2ccc(Br)c(O)c2)c3c(N)ncnc13
Cc1ccc(cc1)n2nc(cc2NC(=O)Nc3ccc(Oc4ccnc5NC(=O)C=Nc45)c6ccccc36)C7(C)CC7
CSc1ccc(cc1)n2nc(cc2NC(=O)Nc3ccc(Oc4ccnc5NC(=O)C=Nc45)c6ccccc36)C(C)(C)C
O=C1Nc2ccccc2/C/1=C/Nc3ccc(cc3)n4cncn4
Oc1ccc2\C(=C\3/C(=O)Nc4ccccc34)\CCc2c1
CNS(=O)(=O)Cc1ccc(cc1)n2nc(cc2NC(=O)Nc3ccc(Oc4ccnc5NC(=O)C=Nc45)c6ccccc36)C(C)(C)C
Fc1ccc(cc1)c2nn3ncccc3c2c4ccnc(Nc5ccc6OCCOc6c5)n4
CC(C)n1nc(c2ccc(Cl)c(O)c2)c3c(N)ncnc13
CCN1CCN(CC1)c2cc(Nc3ncc(s3)c4ccc(NC(=O)Nc5cc(CC)on5)cc4)nc(C)n2

CCc1cccc(NC(=O)Nc2ccc(Oc3ccc4nc(NC(=O)OC)[nH]c4c3)cc2)c1
C(Oc1ccc(Nc2ncnc3ccc(cc23)c4occc4)cc1)c5ccccc5
CC(C)(C)c1cc(NC(=O)Nc2ccc(Oc3ccnc4NC(=O)C=Nc34)c5ccccc25)n(n1)c6ccc(cc6)S(=O)(=O)C
Cc1ccc(cc1)n2nc(cc2NC(=O)Nc3ccc(Oc4ccnc5NC(=O)C=Nc45)c6ccccc36)C(C)(C)C
CC(C)c1cc(NC(=O)Nc2ccc(Oc3ccnc4NC(=O)C=Nc34)c5ccccc25)n(n1)c6ccc(C)cc6
CC(C)(C)c1cc(NC(=O)Nc2ccc(Oc3ccnc4NC(=O)C=Nc34)c5ccccc25)n(n1)c6ccc(CO)nc6
Fc1ccc(Nc2nccc(n2)c3c(nn4ncccc34)c5ccc(cc5)C(F)(F)F)cc1F
CC(C)(C)c1cc(NC(=O)Nc2ccc(Oc3ccnc4NC(=O)C=Nc34)c(Cl)c2Cl)n(n1)c5ccc(CO)cc5
COc1cc(Nc2c(cnc3cc(OCCCN4CCN(C)CC4)c(OC)cc23)C#N)c(Cl)cc1Cl
CC1=Nc2c(Oc3ccc(NC(=O)Nc4cc(nn4c5ccccc5)C(C)(C)C)c6ccccc36)ccnc2NC1=O
CN(c1ccc(NC(=O)Nc2ccc(OC(F)(F)F)cc2)cc1)c3ccnc(Nc4cccc(c4)S(=O)(=O)N)n3
CC(C)(C)c1cc(NC(=O)Nc2ccc(Oc3ccnc4NC(=O)C=Nc34)c5ccccc25)n(n1)c6ccccc6
Cc1ccc2c(c3ccnc(Nc4cccc(c4)C(F)(F)F)n3)c(nn2n1)c5ccccc5
CC(C)(C)c1cc(NC(=O)Nc2ccc(Oc3ccnc4NC(=O)Nc34)c5ccccc25)n(n1)c6ccccc6
Cc1ccc2c(c3ccnc(Nc4ccccc4)n3)c(nn2n1)c5ccc(F)cc5
CN1C(=O)Nc2nccc(Oc3ccc(NC(=O)Nc4cc(nn4c5ccc(C)cc5)C(C)(C)C)c6ccccc36)c12
COc1cc(ccc1NC(=O)OC(C)(C)C)c2nn(C(C)C)c3ncnc(N)c23
Cc1cc(Nc2ncc(s2)C(=O)Nc3c(C)cccc3Cl)nc(C)n1

4. 3ERD

[H][C@](CC)(C1=CC=C(O)C=C1)[C@]([H])(CC)C1=CC=C(O)C=C1
[H][C@]1(C)C=C(C)[C@]2([H])[C@]([H])(C)[C@@]1(CO)CO[C@]2([H])C1=CC=C(O)C=C1
[H][C@]1(C)CC[C@]2(CO)CO[C@]([H])(C3=CC=C(O)C=C3)[C@@]1([H])[C@@]2([H])C
[H][C@]1(O)CC[C@@]2([H])[C@]3([H])CCC4=CC(O)=CC=C4[C@@]3([H])[C@@]([H])(COC)C[C@]12C
[H][C@]1(O)CC[C@@]2([H])[C@]3([H])CCC4=CC(OC(=O)C5=CC=CC=C5)=CC=C4[C@@]3([H])CC[C@]12C
[H][C@@](C)(COC1=CC=C(C=C1)[C@]1([H])OC2=C(S[C@]1([H])C1=CC=C(O)C=C1)C=C(O)C=C2)N1CCCC1
[H][C@@]1(CC[C@@]2([H])[C@]3([H])CCC4=CC(O)=CC=C4[C@@]3([H])CC[C@]12C)OC(=O)CCC1CCCC1
[H][C@@]12C[C@@](CO)(CO[C@]1([H])C1=CC=C(O)C=C1)CC=C2C
[H][C@@]12C[C@@H](O)[C@H](O)[C@@]1(C)CC[C@]1([H])C3=C(CC[C@@]21[H])C=C(O)C=C3
[H][C@@]12CC(F)(F)C[C@]1([H])[C@@]([H])(OC1=C2C=C(O)C=C1)C1=CC=C(O)C=C1
[H][C@@]12CC[C@](C)(O)[C@@]1(C)C[C@H](O)[C@@]1(F)[C@@]2([H])CCC2=CC(=O)CC[C@]12C
[H][C@@]12CC[C@](C)(O)[C@@]1(C)CC[C@@]1([H])[C@@]2([H])CCC2=CC(=O)CC[C@]12C
[H][C@@]12CC[C@](OC(C)=O)(C(C)=O)[C@@]1(C)CC[C@@]1([H])[C@@]2([H])C[C@H](C)C2=CC(=O)CC[C@]12C
[H][C@@]12CC[C@@](C)(O)[C@@]1(C)CCC1=C2C=CC2=CC(O)=CC=C12
[H][C@@]12CC[C@@](O)(C#C)[C@@]1(C)CC[C@]1([H])C3=C(CC(=O)CC3)C[C@@]([H])(C)[C@@]21[H]
[H][C@@]12CC[C@@](O)(C#C)[C@@]1(C)CC[C@]1([H])C3=C(CC[C@@]21[H])C=C(O)C=C3
[H][C@@]12CC[C@@](O)(C#C)[C@@]1(C)CC[C@]1([H])C3=C(CC[C@@]21[H])C=C(OC)C=C3

[H][C@@]12CC[C@@](O)(C#C)[C@@]1(C)CC[C@]1([H])C3=C(CC[C@@]21[H])CC(=O)CC3
[H][C@@]12CC[C@@](O)(C#C)[C@@]1(C)CC[C@@]1([H])[C@@]2([H])CCC2=CC3=C(C[C@]12C)C=NO3
[H][C@@]12CC[C@@](O)(C#C)[C@@]1(CC)C=CC1=C3CCC(=O)C=C3CC[C@@]21[H]
[H][C@@]12CC[C@@](O)(C#C)[C@@]1(CC)CC(=C)[C@]1([H])[C@@]3([H])CCC(=O)C=C3CC[C@@]21[H]
[H][C@@]12CC[C@@](O)(C#C)[C@@]1(CC)CC(=C)[C@]1([H])[C@@]3([H])CCCC=C3CC[C@@]21[H]
[H][C@@]12CC[C@@](O)(C#C)[C@@]1(CC)CC[C@]1([H])[C@@]3([H])CCC(=O)C=C3CC[C@@]21[H]
[H][C@@]12CC[C@@](O)(CC=C)[C@@]1(C)CC[C@]1([H])[C@@]3([H])CCCC=C3CC[C@@]21[H]
[H][C@@]12CC[C@@](OC(C)=O)(C#C)[C@@]1(C)CC[C@]1([H])[C@@]3([H])CC[C@H](OC(C)=O)C=C3CC[C@@]21[H]
[H][C@@]12CC[C@@](OC(C)=O)(C#C)[C@@]1(CC)CC[C@]1([H])[C@@]3([H])CC\C(C=C3CC[C@@]21[H])=N/O
[H][C@@]12CC[C@H](C(C)=O)[C@@]1(C)CC[C@@]1([H])[C@@]2([H])CCC2=CC(=O)CC[C@]12C
[H][C@@]12CC[C@H](O)[C@@]1(C)CC[C@]1([H])C3=C(CC[C@@]21[H])C=C(O)C=C3
[H][C@@]12CC[C@H](O)[C@@]1(C)CC[C@]1([H])C3=CC=C(OC(C)=O)C=C3CC[C@@]21[H]
[H][C@@]12CC[C@H](O)[C@@]1(C)CC[C@@]1([H])[C@@]2([H])CC[C@@]23O[C@@H]2C(O)=C(C[C@]13C)C#N
[H][C@@]12CC[C@H](OC(=O)CCCC)[C@@]1(C)CC[C@]1([H])C3=C(CC[C@@]21[H])C=C(O)C=C3
[H][C@@]12CC[C@H](OC(=O)CCCCCC)[C@@]1(C)CC[C@@]1([H])[C@@]2([H])CCC2=CC(=O)CC[C@]12C
[H][C@@]12CCC(=O)[C@@]1(C)CC[C@]1([H])C3=C(CC[C@@]21[H])C=C(O)C=C3
[H][C@@]12CCC(=O)[C@@]1(C)CC[C@@]1([H])[C@@]2([H])CC=C2C[C@@]([H])(O)CC[C@]12C
[H]\C(CC)=C(/[H])C1=C(O)C=C2CC[C@@]3([H])[C@]4([H])CC[C@]([H])(O)[C@@]4(C)CC[C@]3([H])C2=C1
C[C@]12CC[C@H]3[C@@H](CCC4=C3C=CC(OS(O)(=O)=O)=C4)[C@@H]1CCC2=O
C[C@]12CC[C@H]3[C@@H](CCC4=CC(=O)CC[C@]34C)[C@@H]1CC[C@@H]2OC(=O)CCC1CCCC1
CC(CCC1=CC=C(O)C=C1)NCCC1=CC(O)=C(O)C=C1
CC\C(=C(\CC)C1=CC=C(O)C=C1)C1=CC=C(O)C=C1
CC=C(C(=CC)C1=CC=C(O)C=C1)C1=CC=C(O)C=C1
CC1(C)C(C=C(Cl)Cl)C1C(=O)OCC1=CC(OC2=CC=CC=C2)=CC=C1
CC1=CC=C(\C=C2/C3CCC(C)(C2=O)C3(C)C)C=C1
CCCCCCC(=O)O[C@H]1CC[C@H]2[C@@H]3CCC4=CC(OC(=O)CCCCCC)=CC=C4[C@H]3CC[C@]12C
CN1C(N)=NC2=C1C=C(C=N2)C1=CC=C(O)C=C1
COC1=CC(O)=C(C=C1)C(=O)C1=CC=CC=C1
COC1=CC2=C(NC=C2CCNC(C)=O)C=C1
OC1=CC=C(\C=C\C2=CC(O)=CC(O)=C2)C=C1
OC1=CC=C(C=C1)C1=CC2=C(O1)C(CC#N)=CC(O)=C2
OC1=CC=C(C=C1)N1N=C2C=CC(O)=CC2=C1Cl
OC1=CC=C2C[C@H]3N(CC=C)CC[C@@]45[C@@H](OC1=C24)C(=O)CC[C@@]35O

5. 3LN1

CCCc1ccc(cc1)C2=C(OC(C)(C)C2=O)c3ccc(cc3)S(=O)(=O)C
CC(SC1=Nc2ccccc2C(=O)N1N)C(=O)O
C[S+]([O-])c1ccc(\C=C\2/C=C(CC(=O)O)c3cc(F)ccc23)cc1

COc1ccc(cc1)c2c([nH]c3ccccc23)c4ccc(cc4)S(=O)(=O)N
CC1(C)OC(=C(C1=O)c2cccc(c2)C(F)(F)F)c3ccc(cc3)S(=O)(=O)N
CC(C)(C)Oc1ccc(\C=C\2/C=C(CC(=O)O)c3cc(F)ccc23)cc1
CC(=O)c1ccc(cc1)C2=C(OC(C)(C)C2=O)c3ccc(cc3)S(=O)(=O)N
CCC1(C)OC(=C(C1=O)c2ccc(cc2)C(=O)C)c3ccc(cc3)S(=O)(=O)N
CC1(C)OC(=C(C1=O)c2cccc(Cl)c2)c3ccc(cc3)S(=O)(=O)C
CC1(C)OC(=C(C1=O)c2cc(F)cc(F)c2)c3ccc(c(Cl)c3)S(=O)(=O)N
CC1(C)OC(=C(C1=O)c2cc(F)cc(F)c2)c3ccc(cc3F)S(=O)(=O)N
COc1ccc(cc1)c2c([nH]c3ccccc23)c4ccc(cc4)S(=O)(=O)C
CC1(C)OC(=C(C1=O)c2cccc(F)c2)c3ccc(cc3F)S(=O)(=O)N
CC(C)(C(=O)O)c1cccc(Oc2ccccc2)c1
COc1ccc(C2=C(OC(C)(C)C2=O)c3ccc(cc3)S(=O)(=O)N)c(OC)c1
CC1(C)OC(=C(C1=O)c2cccc(Cl)c2)c3ccc(c(F)c3)S(=O)(=O)N
CC(=O)c1ccc(cc1)C2=C(OC(C)(C)C2=O)c3ccc(cc3)S(=O)(=O)N
CC1(C)OC(=C(C1=O)c2ccc(F)cc2)c3ccc(c(Cl)c3)S(=O)(=O)C
CC1(C)OC(=C(C1=O)c2ccc(cc2)c3ccccc3)c4ccc(cc4)S(=O)(=O)C
CC1(C)OC(=C(C1=O)c2ccccc2F)c3ccc(cc3)S(=O)(=O)N
CC1(C)OC(=C(C1=O)c2cccc(Cl)c2)c3ccc(cc3)S(=O)(=O)N
C[C@@H](C(=O)O)c1ccc(c(F)c1)c2ccccc2
CC1(C)OC(=C(C1=O)c2cccc(Cl)c2)c3ccc(cc3F)S(=O)(=O)C
CCSc1ccc(cc1)C2=C(OC(C)(C)C2=O)c3ccc(cc3)S(=O)(=O)N
CCCCc1ccc(cc1)C2=C(OC(C)(C)C2=O)c3ccc(cc3)S(=O)(=O)C
CS(=O)(=O)c1ccc(cc1)c2[nH]c3ccccc3c2c4ccc(F)cc4
Cc1ccc2[nH]c(c3ccc(cc3)S(=O)(=O)N)c(c4ccccc4)c2c1
CCC1(C)OC(=C(C1=O)c2cccc(OC)c2)c3ccc(cc3)S(=O)(=O)N
CC1(C)OC(=C(C1=O)c2ccccc2)c3ccc(cc3)S(=O)(=O)N
CC1(C)OC(=C(C1=O)c2cccc(F)c2)c3ccc(cc3)S(=O)(=O)C
CC1(C)OC(=C(C1=O)c2ccc(F)c(F)c2)c3ccc(c(Cl)c3)S(=O)(=O)C
CC1(C)OC(=C(C1=O)c2cc(F)cc(F)c2)c3ccc(c(F)c3)S(=O)(=O)N
CC(=O)c1cccc(OC(=O)c2c3CCCCc3sc2c4ccccc4)c1
CC(C)(C(=O)O)C1=C\C(=C/c2ccc(cc2)c3ccccc3)\c4ccc(F)cc14
CC1(C)OC(=C(C1=O)c2cccc(Cl)c2)c3ccc(c(Cl)c3)S(=O)(=O)C
CC1(C)OC(=C(C1=O)c2ccc(Cl)c(Cl)c2)c3ccc(cc3)S(=O)(=O)N
Cc1ccc(cc1)c2cc(nn2c3ccc(cc3)S(=O)(=O)N)C(F)(F)F
CC1(C)OC(=C(C1=O)c2ccc(F)c(F)c2)c3ccc(cc3)S(=O)(=O)N
CC(C)Cc1ccc(cc1)C(C)(C)C(=O)O
CC1(C)OC(=C(C1=O)c2ccc(O)cc2)c3ccc(cc3)S(=O)(=O)N
CC1(C)OC(=C(C1=O)c2cccc(F)c2)c3ccc(cc3)S(=O)(=O)C
CC1(C)OC(=C(C1=O)c2cccc(F)c2)c3ccc(cc3)S(=O)(=O)N
CC1(C)OC(=C(C1=O)c2cc(Cl)cc(Cl)c2)c3ccc(cc3)S(=O)(=O)N

6. 1BNU

CC(C1=CC=C(S(NC2=CC=CC=C2)(=O)=O)C=C1)=O
CC(N(S(C1=CC=CC=C1)(=O)=O)C1SC(S(=O)(=O)N)=NN=1)C
CC(NC1=C(F)C(S(=O)(=O)N)=C(F)C(F)=C1S(CCC1=CC=CC=C1)(=O)=O)(C)C
CC1=CC=C(CN(S(C2=CC=C(C)C=C2)(=O)=O)C2SC(S(=O)(=O)N)=NN=2)C=C1
CC1=CC=C(S(N(C2SC(S(=O)(=O)N)=NN=2)C(C)C)(=O)=O)C=C1
CC1=CC=C(S(N(C2SC(S(=O)(=O)N)=NN=2)C)(=O)=O)C=C1
CC1=CC=C(S(N(C2SC(S(=O)(=O)N)=NN=2)CC(C)C)(=O)=O)C=C1
CC1=CC=C(S(N(C2SC(S(=O)(=O)N)=NN=2)CC)(=O)=O)C=C1
CC1=CC=C(S(N(C2SC(S(=O)(=O)N)=NN=2)CC2=CC=C(Cl)C=C2)(=O)=O)C=C1
CC1=CC=C(S(N(C2SC(S(=O)(=O)N)=NN=2)CC2=CC=C(F)C=C2)(=O)=O)C=C1
CC1=CC=C(S(N(C2SC(S(=O)(=O)N)=NN=2)CC2=CC=C(OC)C=C2)(=O)=O)C=C1
CC1=CC=C(S(N(C2SC(S(=O)(=O)N)=NN=2)CC2=CC=CC=C2)(=O)=O)C=C1
CC1=CC=C(S(N(C2SC(S(=O)(=O)N)=NN=2)CC2C(Cl)=CC(Cl)=CC=2)(=O)=O)C=C1
CC1=CC=C(S(N(C2SC(S(=O)(=O)N)=NN=2)CC2C=C(OC)C(OC)=C(OC)C=2)(=O)=O)C=C1
CC1=CC=C(S(N(C2SC(S(=O)(=O)N)=NN=2)CCC)(=O)=O)C=C1
CC1=CC=C(S(N(C2SC(S(=O)(=O)N)=NN=2)CCC2=CC=C(Br)C=C2)(=O)=O)C=C1
CC1=CC=C(S(N(C2SC(S(=O)(=O)N)=NN=2)CCC2=CC=CC=C2)(=O)=O)C=C1
CC1=CC=C(S(N(C2SC(S(=O)(=O)N)=NN=2)CCCC)(=O)=O)C=C1
CC1=CC=C(S(N(C2SC(S(=O)(=O)N)=NN=2)CCCC2C(OC)=CC=CC=2)(=O)=O)C=C1
CC1=CC=C(S(N(C2SC(S(=O)(=O)N)=NN=2)CCCCC)(=O)=O)C=C1
CC1C=CC(S(NCC2=CC=CC=C2)(=O)=O)=CC=1
CCCCCN(S(C1=CC=CC=C1)(=O)=O)C1SC(S(=O)(=O)N)=NN=1
CCCCN(S(C1=CC=CC=C1)(=O)=O)C1SC(S(=O)(=O)N)=NN=1
CCCN(S(C1=CC=CC=C1)(=O)=O)C1SC(S(=O)(=O)N)=NN=1
CCN(S(C1=CC=CC=C1)(=O)=O)C1SC(S(=O)(=O)N)=NN=1
CCOC(C1C(C(NC2SC(S(=O)(=O)N)=NN=2)=O)=NN(C2C=CC(N)=CC=2)C=1C1=CC=CC=C1)=O
CCOC(C1N=NN(C2=CC=C3C(C=CS(O3)(=O)=O)=C2)C=1)=O
CN(S(C1=CC=CC=C1)(=O)=O)C1SC(S(=O)(=O)N)=NN=1
CN(S(C1SC(N(S(C2=CC=C(C)C=C2)(=O)=O)C)=NN=1)(=O)=O)C
COC1=CC=C(C2N=NN(C3=CC=C4C(C=CS(O4)(=O)=O)=C3)C=2)C=C1
COC1=CC=C(CC(N2CCN(C(NC3=CC=C(S(=O)(=O)N)C=C3)=O)CC2)=O)C=C1
COC1=CC=C(CN(S(C2=CC=CC=C2)(=O)=O)C2SC(S(=O)(=O)N)=NN=2)C=C1
COC1=CC=CC(C2N=NN(C3=CC=C4C(C=CS(O4)(=O)=O)=C3)C=2)=C1
COC1C(O)=C(OC)C=C(CC(N2CCN(C(NC3=CC=C(S(=O)(=O)N)C=C3)=O)CC2)=O)C=1
NS(C1=CC=C(C(NCCCCCNC(C2=CC=CC(S(OCCOCCOC3=CC(C(F)(F)F)=CC(C(F)(F)F)=C3)(=O)=O)=C2)=O)=O)C=C1)(=O)=O
NS(C1=CC=C(C2=C(Cl)SC(NC(N(CCN3CCOCC3)CCC(C3=CC=C(F)C=C3)C3=CC=C(F)C=C3)=O)=N2)C=C1)(=O)=O
NS(C1=CC=C(NC(N2CCN(C(COC3=CC=C(Cl)C=C3)=O)CC2)=O)C=C1)(=O)=O
NS(C1=CC=C(NC(N2CCN(C(COC3=CC=CC=C3)=O)CC2)=O)C=C1)(=O)=O
NS(C1=CC=C(NC(N2CCN(C(COC3C(Cl)=CC(Cl)=CC=3)=O)CC2)=O)C=C1)(=O)=O

NS(C1C=CC(NC(N2CCN(C(CC3=CC=C(Cl)C=C3)=O)CC2)=O)=CC=1)(=O)=O
NS(C1C=CC(NC(N2CCN(C(CC3=CC=C(O)C=C3)=O)CC2)=O)=CC=1)(=O)=O
NS(C1OC2=C(C=C(S2)CNCC2N=CC=CC=2)C=1)(=O)=O
NS(C1SC(N(S(C2=CC=CC=C2)(=O)=O)CC2=CC=C(Cl)C=C2)=NN=1)(=O)=O
NS(C1SC(N(S(C2=CC=CC=C2)(=O)=O)CC2=CC=CC=C2)=NN=1)(=O)=O
NS(C1SC(N(S(C2=CC=CC=C2)(=O)=O)CC2C(Cl)=CC(Cl)=CC=2)=NN=1)(=O)=O
NS(C1SC(N(S(C2=CC=CC=C2)(=O)=O)CCC2=CC=C(Br)C=C2)=NN=1)(=O)=O
NS(C1SC(N(S(C2=CC=CC=C2)(=O)=O)CCC2=CC=CC=C2)=NN=1)(=O)=O
NS(C1SC2=C([C@H](CCS2(=O)=O)O)C=1)(=O)=O
O=C(C1=CC=CC=C1)CSC1SC(SCC(C2=CC=CC=C2)=O)=NN=1
O=C(C1=CC=CC=C1)NS(C1=CC=CC=C1)(=O)=O
O=S(N(C1SC(S(N(CC2=CC=CC=C2)CC2=CC=CC=C2)(=O)=O)=NN=1)CC1=CC=CC=C1)(C1=CC=CC=C1)=O
O=S1(OC2=CC=C3C(=C2C=C1)C=CC=C3)=O
O=S1(OC2C(=CC(N3N=NC(C4=CC=C(OC(F)(F)F)C=C4)=C3)=CC=2)C=C1)=O
O=S1(OC2C(=CC(N3N=NC(C4=CC=CC=C4)=C3)=CC=2)C=C1)=O
O=S1(OC2C(=CC(N3N=NC(C4C=CC=C(C(F)(F)F)C=4)=C3)=CC=2)C=C1)=O
OC([C@@H](NS(C1=CC=C(C2=CC=CC=C2)C=C1)(=O)=O)CC1=CC=CC=C1)=O
OC1=CC=CC(C(CSC2OC(C3=CC=C(Cl)C=C3)=NN=2)=O)=C1
OC1=CC=CC(C(CSC2SC(SCC(C3=CC=CC(O)=C3)=O)=NN=2)=O)=C1
ON1C(=O)C=CC=C1C(NCC1=CC=C(C2=CC=CC=C2)C=C1)=O
ONC(/C(/C(NCC1=CC=C(F)C=C1)=O)=C\C1=CC=CC=C1)=O

7. 1B8O

CC(C)c1ccc(Cc2c[nH]c3C(=O)NC(=Nc23)N)cc1
CC1CCCC(Cc2c[nH]c3c(O)nc(N)nc23)C1
Cc1nc2c(O)[nH]c(N)nc2c1Cc3ccccc3
CCOP(=O)(OCC)C(F)(F)CCCCCc1c[nH]c2C(=O)NC(=Nc12)N
CCOP(=O)(OCC)C(F)(F)CCCCCCCc1c[nH]c2C(=O)NC(=Nc12)N
COc1cccc(Cc2c[nH]c3c(O)nc(N)nc23)c1
NC(=O)c1[nH]nc(C2N[C@H](CO)[C@H](O)[C@@H]2O)c1N
NC1=NC(=O)C2NC=C(Cc3ccccc3)C2N1
NC1=Nc2c(c[nH]c2C(=O)N1)[C@@H]3N[C@H](CO)[C@@H](O)[C@H]3O
NC1=Nc2c(Cc3ccc(OCc4ccccc4)cc3)c[nH]c2C(=O)N1
NC1=Nc2c(Cc3cccc(Cl)c3)c[nH]c2C(=O)N1
NC1=Nc2c(Cc3cccnc3)c[nH]c2C(=O)N1
NC1=Nc2c(CCCCC(F)(F)P(=O)(O)O)c[nH]c2C(=O)N1
NC1=Nc2c(CCCCCC(F)(F)P(=O)(O)O)c[nH]c2C(=O)N1
NC1=Nc2c(ncn2CCCCC(F)(F)P(=O)(O)O)C(=O)N1
Nc1nc(O)c2[nH]c(N)c(CC3CCCCC3)c2n1
Nc1nc(O)c2[nH]cc([C@@H](CC(=O)O)c3cccc(Cl)c3)c2n1

Nc1nc(O)c2[nH]cc(C(CC(=O)O)c3ccccc3)c2n1
Nc1nc(O)c2[nH]cc(C(CC#N)c3cccc(Cl)c3)c2n1
Nc1nc(O)c2[nH]cc(C3=CCCCC3)c2n1
Nc1nc(O)c2[nH]cc(C3CCCCC3)c2n1
Nc1nc(O)c2[nH]cc(CC3C4CC5CC(CC3C5)C4)c2n1
Nc1nc(O)c2[nH]cc(Cc3ccc(cc3)c4ccccc4)c2n1
Nc1nc(O)c2[nH]cc(Cc3ccc(Cl)cc3)c2n1
Nc1nc(O)c2[nH]cc(Cc3ccc(I)cc3)c2n1
Nc1nc(O)c2[nH]cc(Cc3ccc(O)cc3)c2n1
Nc1nc(O)c2[nH]cc(CC3CCCC(C3)C(F)(F)F)c2n1
Nc1nc(O)c2[nH]cc(Cc3cccc(c3)C(F)(F)F)c2n1
Nc1nc(O)c2[nH]cc(Cc3cccc(F)c3)c2n1
Nc1nc(O)c2[nH]cc(Cc3cccc(O)c3)c2n1
Nc1nc(O)c2[nH]cc(Cc3cccc(Oc4ccccc4)c3)c2n1
Nc1nc(O)c2[nH]cc(CC3CCCC3)c2n1
Nc1nc(O)c2[nH]cc(CC3CCCCC3)c2n1
Nc1nc(O)c2[nH]cc(CC3CCCS3)c2n1
Nc1nc(O)c2[nH]cc(Cc3ccsc3)c2n1
Nc1nc(O)c2[nH]cc(Oc3ccccc3)c2n1
Nc1nc(O)c2ncn(Cc3ccccc3\C=C(/F)\P(=O)(O)O)c2n1
Nc1nc(O)c2ncn(Cc3ccccc3\C=C\P(=O)(O)O)c2n1
Nc1nc(O)c2ncn(Cc3ccccc3C(F)C(F)(F)P(=O)(O)O)c2n1
Nc1nc(O)c2ncn(Cc3ccccc3CC(F)(F)P(=O)(O)O)c2n1
OC[C@H]1CN(Cc2c[nH]c3C(=O)NC=Nc23)C[C@@H]1O
OC[C@H]1N[C@H]([C@H](O)[C@@H]1O)c2c[nH]c3C(=O)NC=Nc23
OC[C@H]1N[C@H]([C@H](O)[C@@H]1O)c2n[nH]c3c(O)ncnc23
Oc1[nH]cnc2c(Cc3ccccc3)cnc12
Oc1ncn(Cc2ccccc2)c3ncnc13
OCC1(CO)CN(Cc2c[nH]c3C(=O)NC=Nc23)C1
OCC1CCN1Cc2c[nH]c3C(=O)NC=Nc23
OCC1CN(Cc2c[nH]c3C(=O)NC=Nc23)C1
OP(=O)(O)C(F)(F)C[C@@H]1CCOC[C@@H]1Cn2cnc3C(=O)NC=Nc23

List of Inactives for 1BNU

ONC(=O)CN1CN=C(Cl)C1Cl
CC1=CC(=O)Oc2cc(O[C@@H]3OC[C@@H](O)[C@@H](O)[C@H]3O)ccc12
CC1=CC(=CC(=O)O1)C
CC(C)CN(CC(C)C)S(=O)(=O)N
CC1(C)O[C@H]2[C@@H]3OC4(CCCCC4)O[C@@H]3CO[C@@]2(CNS(=O)(=O)N)O1

OCCOc1ccc2C=CC(=O)Oc2c1
COc1ccc(\C=C\2/NC(=S)NC2=O)c(OC)c1
COc1ccc(\C=C\2/NC(=O)NC2=O)c(OC)c1
C=CCOc1ccc2C=CC(=S)Oc2c1
CC1=CC(=O)Oc2cc(O[C@@H]3O[C@H](CO)[C@@H](O)[C@H](O)[C@H]3O)ccc12
OCl(=O)(=O)=O
COC(=O)CCNS(=O)(=O)NCCC(=O)OC
FCCOc1ccc2C=CC(=O)Oc2c1
C=CCOC1=CC(=O)Oc2ccccc12
CCCCN(CCCC)S(=O)(=O)N(CCCC)CCCC
CCOP(=S)(OCC)Oc1ccc2C(=C(Cl)C(=O)Oc2c1)C
COC(=O)CCNS(=O)(=O)Oc1ccccc1O
COc1ccc(\C=C\2/SC(=O)NC2=O)c(OC)c1
CC1=CC(=O)Oc2cc(O[C@@H]3OC[C@@H](O)[C@H](O)[C@H]3O)ccc12
COc1ccc(CC2NC(=O)NC2=O)c(OC)c1
COC(=O)[C@@H](NS(=O)(=O)N[C@@H](C(C)C)C(=O)OC)C(C)C
[O-][N+](=O)c1ccc2NC(=O)NC(=O)c2c1
O=C1NC(=O)\C(=C\c2ccccc2)\N1
CC1=CC(=O)Oc2cc(NC(=O)OC(C)(C)C)ccc12
CC(C)N(C(C)C)S(=O)(=O)N
CC1(C)O[C@@H]2CO[C@]3(OC(C)(C)O[C@H]3[C@@H]2O1)C(=O)NS(=O)(=O)N
CC1=CC(=O)Oc2cc(N)ccc12
CCCN(CCC)S(=O)(=O)N(CCC)CCC
CC(=O)c1c(OCc2ccccc2)ccc3C=CC(=O)Oc13
C=CCOc1ccc2C=CC(=O)Oc2c1
CC1=CC(=C\C(=N/O)\O1)C
CC1=CC(=CC(=S)O1)C
COC(=O)[C@H](C)NS(=O)(=O)N[C@@H](C)C(=O)OC
C=CCOc1ccc2OC(=O)C=Cc2c1
CC(O)C[Se]c1ccccc1
COc1ccc2C=CC(=O)Oc2c1
COC(=O)[C@H](C)NS(=O)(=O)NC(=O)OC(C)(C)C
CCCCNS(=O)(=O)NCCC(=O)OC
CN(C)S(=O)(=O)NCC1COc2ccccc2O1
FC(F)(F)C(=O)NCCNCCNCCNC(=O)C(F)(F)F
O=S(=O)(NC1CC1)NC2CC2
NS(=O)(=O)c1c(F)c(F)c(SCCc2ccccc2)c(F)c1NC3CCCCCCCCCCC3
CN1C(=O)OC(C)(C)C1=O
CC(C)(C)OC(=O)N(CCCN(CCNC(=O)C(F)(F)F)C(=O)OC(C)(C)C)CCNC(=O)C(F)(F)F
CC1(C)O[C@H]2[C@@H]3OC4(CCCC4)O[C@@H]3CO[C@@]2(CNS(=O)(=O)N)O1
CC(=O)c1c(OCCC23CC4CC(CC(C4)C2)C3)ccc5C=CC(=O)Oc15
ON1C(=S)C=CC=C1C(F)(F)F

CC1(C)OC(=O)NC1=O
CC(C)(C)OC(=O)N(CCNC(=O)C(F)(F)F)CCN(CCNC(=O)C(F)(F)F)C(=O)OC(C)(C)C
Cc1ccc(cc1)S(=O)(=O)OCCOc2ccc3C=CC(=O)Oc3c2
CC1(C)O[C@@H]2CO[C@]3(OC(C)(C)O[C@H]3[C@@H]2O1)C(=O)NNS(=O)(=O)N
C=CCOc1ccc2OC(=S)C=Cc2c1
CC1(C)Oc2cc3OC(=O)C=Cc3cc2C=C1
CC(=O)Nc1ccc2C(=CC(=O)Oc2c1)C
OS(=O)(=O)O
OC(=O)C1=Cc2ccccc2OC1=O
CC1=CC(=O)Oc2cc(NC(=O)c3ccccc3NC(=O)c4ccc(Cl)cc4)ccc12
O=C1NC(Cc2ccccc2)C(=O)N1
CCCN(CCC)S(=O)(=O)NC(=O)OC(C)(C)C
CC1=CC(=O)Oc2cc(NC(=O)Nc3cc(C)cc(C)c3)ccc12

Compounds for Polymorphic Validation - See Table 4 in the Paper

Cc1ccc(cc1)c2cc(nn2c3ccc(cc3)S(=O)(=O)N)C(F)(F)F
C[C@]12CC[C@H]3[C@@H](CCc4cc(OS(=O)(=O)N)ccc34)[C@@H]1CCC2=O
CCOC(=O)c1ccc(O)cc1
Oc1ccc(\C=C\c2cc(O)cc(O)c2)cc1
O\N=C\c1c(O)ccc(c1Cl)c2ccc(O)cc2
O\N=C\c1c(O)ccc(c1Cl)c2ccc(O)c(F)c2
Cc1cn(cn1)c2cc(NC(=O)c3ccc(C)c(Nc4nccc(n4)c5cccnc5)c3)cc(c2)C(F)(F)F
C[C@]12CC[C@H]3[C@@H](CCc4cc(O)ccc34)[C@@H]1CC[C@@H]2OC(=O)[C@@H]5CCCN5C(=O)c6ccc(cc6)S(=O)(=O)N
OC(=O)\C=C\c1ccc(O)cc1

Property Distributions of Known Actives and Inactives for 1BNU

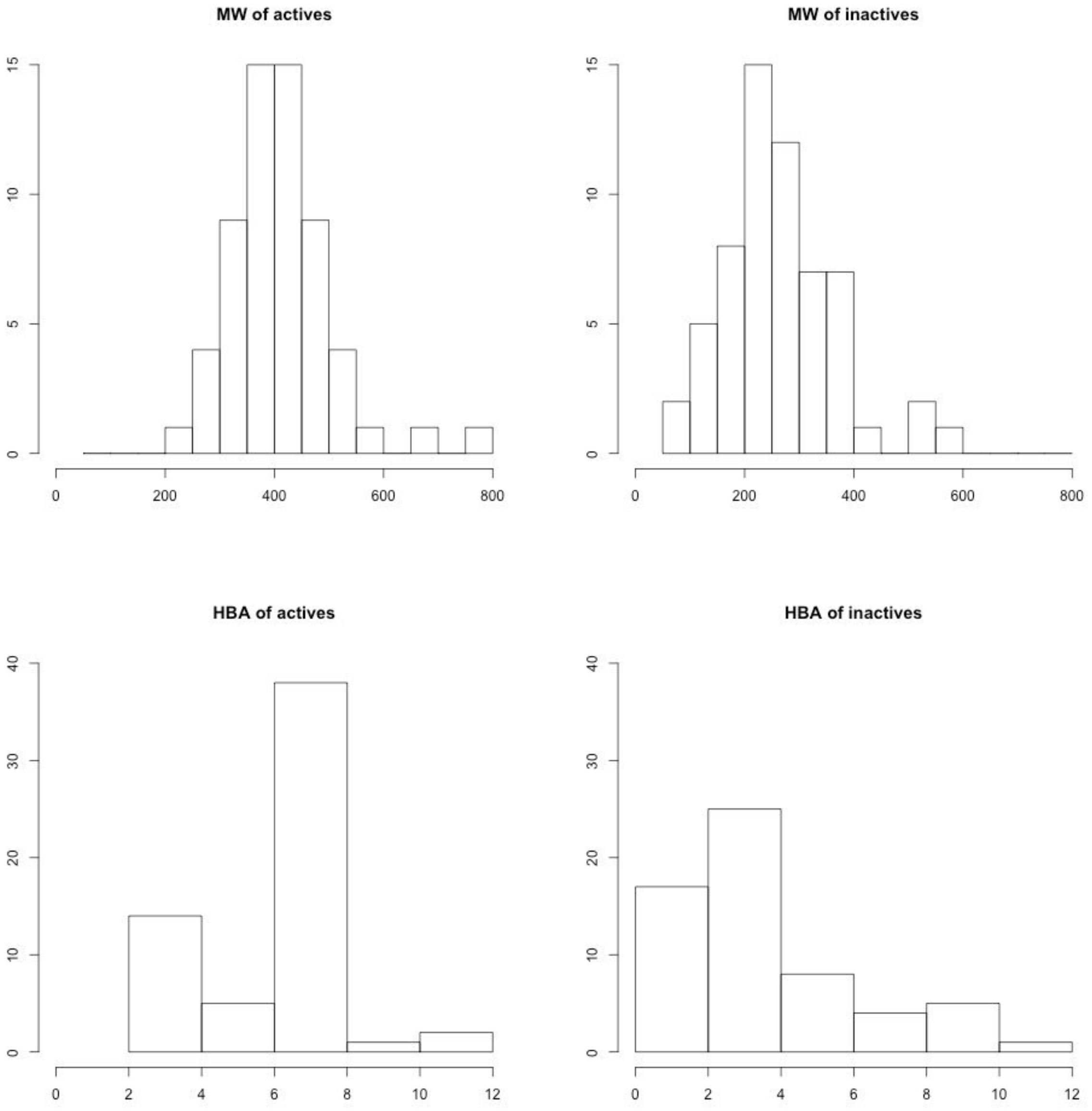

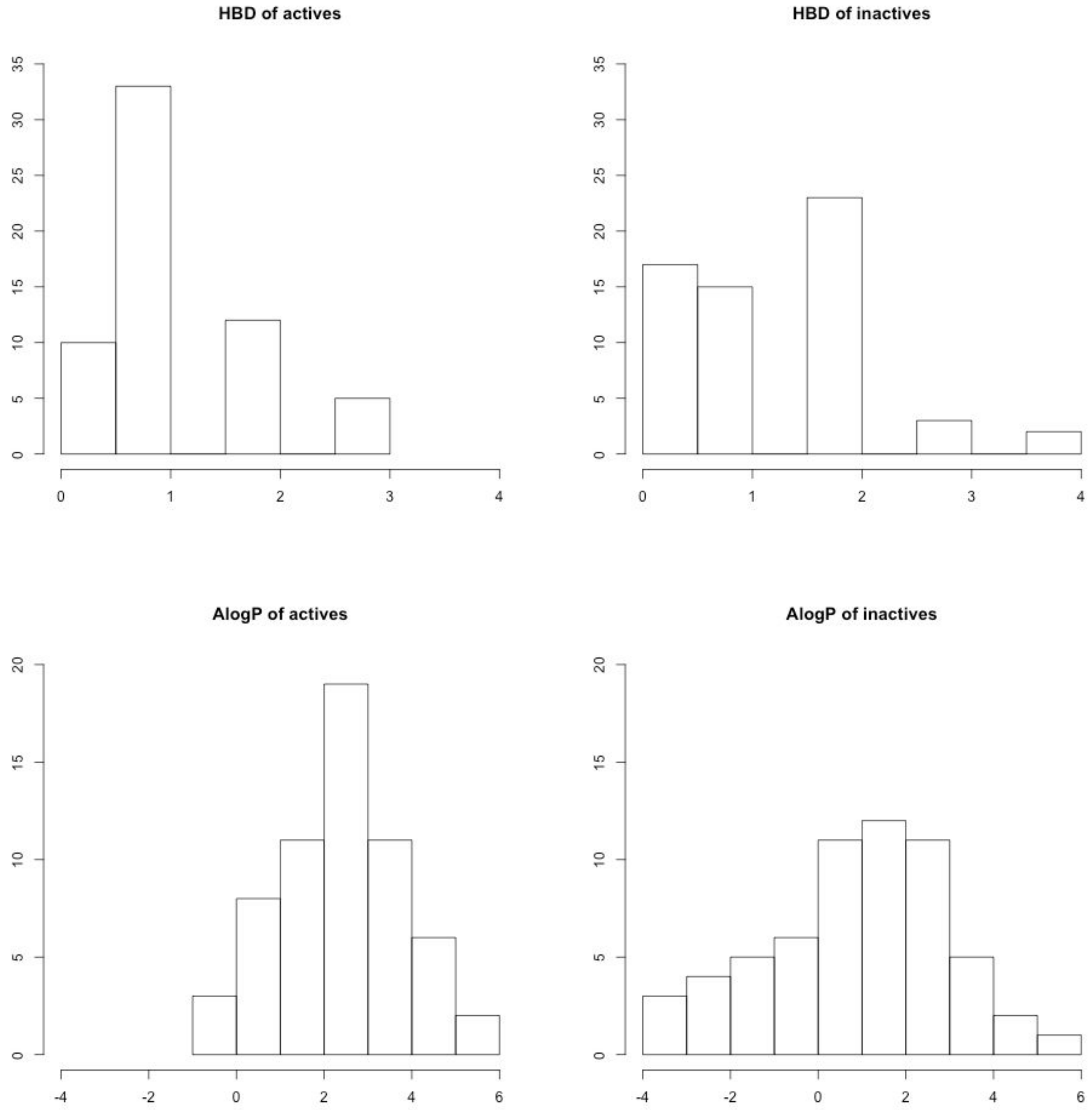
HBD of actives
HBD of inactives
AlogP of actives
AlogP of inactives

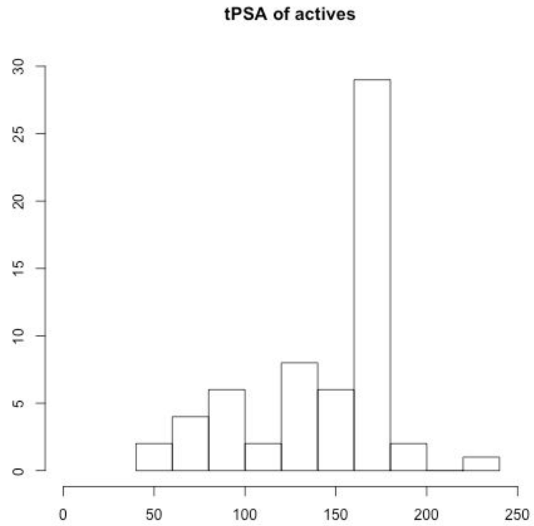
tPSA of actives
30
25
20
15
10
5
0
0
50
100
150
200
250
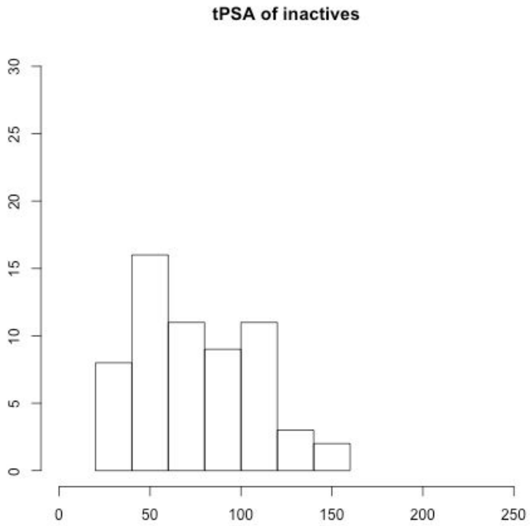
tPSA of inactives
30
25
20
15
10
5
0
0
50
100
150
200
250
